# GABA_*B*_ Receptor-Mediated Regulation of Dendro-Somatic Synergy in Layer 5 Pyramidal Neurons

**DOI:** 10.3389/fncel.2021.718413

**Published:** 2021-08-25

**Authors:** Jan M. Schulz, Jim W. Kay, Josef Bischofberger, Matthew E. Larkum

**Affiliations:** ^1^Department of Biomedicine, University of Basel, Basel, Switzerland; ^2^Department of Statistics, University of Glasgow, Glasgow, United Kingdom; ^3^Institute for Biology, Humboldt-Universität zu Berlin, Berlin, Germany

**Keywords:** neocortex, dendrite, interneuron, neurogliaform cells, GIRK channels, interhemispheric inhibition, L-type calcium channels, HCN channels – h current

## Abstract

Synergistic interactions between independent synaptic input streams may fundamentally change the action potential (AP) output. Using partial information decomposition, we demonstrate here a substantial contribution of synergy between somatic and apical dendritic inputs to the information in the AP output of L5b pyramidal neurons. Activation of dendritic GABA_*B*_ receptors (GABA_*B*_Rs), known to decrease APs *in vivo*, potently decreased synergy and increased somatic control of AP output. Synergy was the result of the voltage-dependence of the transfer resistance between dendrite and soma, which showed a two-fold increase per 28.7 mV dendritic depolarization. GIRK channels activated by dendritic GABA_*B*_Rs decreased voltage-dependent transfer resistances and AP output. In contrast, inhibition of dendritic L-type Ca^2+^ channels prevented high-frequency bursts of APs, but did not affect dendro-somatic synergy. Finally, we show that NDNF-positive neurogliaform cells effectively control somatic AP via synaptic activation of dendritic GIRK channels. These results uncover a novel inhibitory mechanism that powerfully gates cellular information flow in the cortex.

## Introduction

The mammalian cortex is a conspicuously layered structure that receives inputs from afferent subcortical and other cortical areas in a layer-specific manner (Douglas and Martin, 2004). The dendrites of pyramidal neurons typically span several layers placing them in the ideal position to form associative computational elements of different input streams (Larkum, 2013). Layer 5 (L5) pyramidal neurons act as coincidence detectors of sensory feedforward inputs impinging onto the basal dendrites and associative higher-order feedback inputs onto the apical tuft (Xu et al., 2012; Takahashi et al., 2016). The interaction between feedback and feedforward inputs is thought to detect correlations between different input streams by amplifying or attenuating AP output. Apical amplification of AP output probably forms the cellular basis for contextual modulation, crucial to the dynamic coordination of neocortical cell assemblies, and contributes to conscious information processing (Larkum, 2013; Aru et al., 2020; Suzuki and Larkum, 2020). The information in the AP output about the combined inputs is captured by the Mutual Information (Shannon, 1948). Partial Information Decomposition (PID; Williams and Beer, 2010) can be used to estimate the specific contribution of synergy between independent inputs, i.e., information contained in the AP output that exceeds the sum of information of the individual inputs (Gawne and Richmond, 1993; Panzeri et al., 1999; Schneidman et al., 2003; Griffith and Koch, 2014; Timme and Lapish, 2018; Kay and Phillips, 2020).

One important mechanism for apical amplification is probably the generation of dendritic Ca^2+^ spikes by the interaction of backpropagating APs and dendritic postsynaptic potentials (PSP) resulting in bursts of axonal APs (Larkum et al., 1999; Larkum et al., 2004). Recently, however, it was shown that coupling between the apical and basal compartments mainly depends on cholinergic and metabotropic glutamate receptor activation in awake mice, but may be independent of L-type Ca^2+^Channels (Suzuki and Larkum, 2020). This suggests that other cell-intrinsic voltage-dependent mechanisms may contribute to the synergistic interaction between depolarization in the dendritic and perisomatic compartments. Despite the importance of this question, no unbiased approach has been applied yet to tease apart the contributions of Ca^2+^-dependent and independent mechanisms involved in apical amplification.

Active dendritic processing significantly contributes to sensory perception and behaviorally relevant neuronal computations (Xu et al., 2012; Smith et al., 2013; Bittner et al., 2015; Takahashi et al., 2016, 2020; Sheffield et al., 2017). Apart from multiple modulatory systems (Santello and Nevian, 2015; Liu et al., 2017; Labarrera et al., 2018; Williams and Fletcher, 2019), inhibition is a powerful regulator of dendritic integration (Palmer et al., 2012; Cichon and Gan, 2015; Milstein et al., 2015; Schulz et al., 2018). GABA_*B*_ receptors (GABA_*B*_Rs) in particular may play an important role. Dendritic GABA_*B*_Rs mediate a slow form interhemispheric inhibition that decreases the *in vivo* AP rate specifically in L5 pyramidal neurons by about a third without noticeable effects on the subthreshold voltage envelope at the cell body (Palmer et al., 2012, 2013). Although perisomatic GABA_*B*_Rs have been reported to have a larger effect on G protein-coupled inwardly-rectifying K^+^ (GIRK) channel activation, dendritic GABA_*B*_Rs both activate GIRK channels and inhibit dendritic Ca^2+^ channels (Perez-Garci et al., 2006; Breton and Stuart, 2012; Palmer et al., 2012; Perez-Garci et al., 2013). However, the relative contributions of the two GABA_*B*_R- activated effector pathways to the regulation of apical amplification is not well understood.

In the current study, we systematically mapped the effect of combined current injections into the soma and apical dendrite and used PID to elucidate the mechanisms underlying dendro-somatic synergy and its regulation by GABA_*B*_R-activated effector pathways.

## Materials and Methods

### Slice Preparation

Wistar rats (P28-P40) were anesthetized with 95% CO_2_/5% O_2_ before decapitation. The brain was then rapidly transferred to ice-cold, oxygenated artificial cerebrospinal fluid (ACSF) containing (in mM) 125 NaCl, 25 NaHCO_3_, 2.5 KCl, 1.25 NaH_2_PO_4_, 1 MgCl_2_, 25 glucose and 2 CaCl_2_ (pH 7.4). Parasagittal slices of the primary somatosensory cortex (300 μm thick) were cut with a vibrating microslicer (Leica) and incubated at 37°C for 30 min and subsequently maintained at room temperature (∼22°C). Dual somatic and dendritic whole-cell patch recordings were made from visually identified neurons using infrared Dodt gradient contrast or oblique illumination and a CCD camera (CoolSnap ES, Roper Scientific). During recordings, slices were bathed in ACSF maintained at 33–35°C.

In addition, the following transgenic mouse lines were used: SOM-Cre [SST tm2.1(cre)Zjh/J], NDNF-Cre [C57BL/6-Ndnf tm(cre/ERT2)] generated in Ivo Spiegel’s lab (Abs et al., 2018), and LoxP-ChR2 [B6.Cg-Gt(ROSA)26Sortm32(CAG-COP4^∗^H134R/EYFP)Hze/J]. NDNF-Cre X flox-ChR2 mice received intraperitoneal injections of tamoxifen (0.12 mg/g body weight, dissolved in 90% corn oil and 10% ethanol at 20 mg/ml) on 2 consecutive days 2–4 weeks before the actual experiment. In order to increase cell viability, mice were exposed to oxygen-enriched atmosphere for 10 min prior anesthesia with isoflurane (4% in O_2_, Vapor, Draeger) and decapitation, in accordance with national and institutional guidelines. In these experiments, a sucrose-based solution was used, containing 87 NaCl, 25 NaHCO_3_, 2.5 KCl, 1.25 NaH2PO_4_, 75 sucrose, 0.5 CaCl_2_, 7 MgCl_2_ and 10 glucose (equilibrated with 95% O_2_/5% CO_2_) for slice preparation cutting and storage.

### Patch-Clamp Recordings

Whole-cell patch-clamp recordings were obtained from thick-tufted layer 5b (L5) pyramidal neurons using a patch pipette (resistance 6–9 MΩ) filled with an intracellular solution containing (in mM): 135 K gluconate, 4 KCl, 10 mM HEPES, 10 Na_2_ phosphocreatine, 4 Mg-ATP, 0.3 Na-GTP, 0.2% biocytin, adjusted to pH 7.3-7.4 with NaOH. In addition, pipettes targeting the soma contained the fluorescent dye Alexa 594 (Molecular Probes, 10 μM) for visualizing the dendritic tree during the experiments. Dual whole-cell voltage recordings were performed from the soma and dendrite (resistance 20–40 MΩ) using Axoclamp 2A (Molecular Devices) and Dagan BVC-700A amplifiers (Dagan Corporation). Voltage was filtered at 5 kHz and digitized at 10 kHz using a BNC900 (National Instruments, Austin, TX, United States) or ITC-18 interface (InstruTech, Port Washington, NY, United States). Custom written igor software was used for acquisition. No correction was made for the junction potential between the bath and pipette solutions. Dual recordings were made from the soma and dendrite in current clamp mode. Current of varying amplitude was injected through either one or both electrodes simultaneously. Step currents were 1 s long, and in other experiments, two different voltage responses to contralateral-HS recorded from the soma and dendrite *in vivo* (Palmer et al., 2012) were injected into the soma and dendrite, respectively.

### Optogenetic Stimulation

A diode laser (DL-473, Rapp Optoelectronic) was coupled to the epifluorescent port of the microscope (Zeiss Examiner, equipped with a 63× NA1.0 water immersion objective; Carl Zeiss Microscopy GmbH, Jena, Germany) via fiber optics. The laser was controlled via TTL pulses. For the optogenetic activation of the axon of specific interneuron subpopulations, the field of view was shifted, such that the most distal portion of the apical dendrite of the recorded neuron was inside the illuminated area, using laser intensities of up to 9 mW for 5 ms.

### Drugs Applications

Baclofen (50 μM) was pressure ejected from a glass pipette (tip diameter: 1 μm) placed 50–100 μm distal to the dendritic patch pipette (approx. 500–800 μm from the soma). The volume directly affected by the drugs pressure ejected from the puffing pipette was estimated to have a radius of approx. 100 μm, as measured by a test application of the fluorescent indicator Alexa 594 into a brain slice. The GABA_*B*_R antagonist CGP 54626 hydrochloride (Tocris) was dissolved in DMSO (10 mM). Nimodipine (Sigma) was dissolved at 20 mM in DMSO on the day of the experiment. In experiments testing GIRK channel contribution, tertiapin (Sigma) was added to the bath ACSF (0.5 μM) and puff pipette (5.0 μM).

### Neuronal Reconstruction

After recordings, neurons were prepared for biocytin reconstruction. The slices were placed in 4% paraformaldehyde after the experiment for up to 4 days. Slices were processed for biocytin staining to reveal the morphology of the recorded neuron. Neuronal reconstruction was then performed with Neurolucida software.

### Immunohistochemistry

After electrophysiological recordings, brain slices were fixed in 4% paraformaldehyde overnight. Washing was done with a step-wise protocol using a tris-buffered saline, and 0.3% triton solution. Slices were transferred to the same tris-buffered saline containing donkey serum (5%) for 2 h to block unspecific binding of antibodies. Incubation with a primary anti-GFP antibody (1:1000; polyclonal chicken, Abcam) in 5% donkey serum was done for 72 h at 4°C. Subsequently, slices were rinsed with tris-buffered saline and incubated with donkey-anti-chicken-Alexa Fluor 488 (1:500; Invitrogen) and Alexa Fluor 568 conjugated Streptavidin (1:1000; MoBiTec) for 72 h at 4°C. After the final rinsing, slices were mounted with ProLong Gold Antifade (Invitrogen), and imaged using a Zeiss LSM900 confocal microscope (Oberkochen, Germany). Image analysis and processing was done using the Zeiss ZEN software and ImageJ freeware^1^.

### Data Analysis

Data were analyzed offline using MATLAB 7.13 with Signal Processing 6.16 and Statistics 7.6 Toolboxes. Action potentials (APs) were detected using a threshold criterion when the membrane potential crossed 0 mV. The time of the maximal depolarization was saved for each AP. The spike rate was defined as number of APs during the 1 s-long current step. Interspike intervals (ISI) were calculated as the difference in time between two subsequent APs. For experiments with fluctuating current stimuli, segments of the injected current waveform around the time of each AP were saved for visualization. APs were classified into three categories: single APs (preceding ISI_*n*__–__1_ and subsequent ISI_*n*_ > 15 ms), APs at the start of a burst (ISI_*n–1*_ > 15 ms and ISI_*n*_ < 15 ms), and APs during a burst (ISI_*n–1*_ < 15 ms). The spike-triggered average (STA) was calculated as the mean of these current segments over all spikes of a defined class. For the analysis of the baclofen-induced effect on trials with low spike rates (Figure 7F), only episodes with a maximal dendritic current amplitude of 750 pA and less than 7 spikes in control were included.

Input and transfer resistances were determined from the slope of a regression line fitted to four mean membrane potentials produced by a series of subthreshold current pulses around resting membrane potential (−100, 0, +100, and +200 pA). For transfer resistances, current was injected into either somatic or dendritic electrode and membrane potentials were measured at the opposite electrode. For experiments involving tertiapin application, input and transfer resistances were directly derived from 250 pA current injections into dendrite and soma, respectively. To test for a voltage-dependence of the input (transfer) resistance, input (transfer) resistances were determined for increasing current step amplitudes according to Ohm’s law and then normalized by the input (transfer) resistances measured at rest. The dependence of input (transfer) resistance on membrane potential or current level was determined by fitting an exponential curve [y = *Y0*^∗^e(*k*^∗^*x*)] to the data. For this analysis, only experiments with optimal compensation of series resistances were included.

#### Partial Information Decomposition

Partial information decomposition (Williams and Beer, 2010) is a method for splitting the joint mutual information *I*(*Y*; *S*, *D*) into four non-negative components:

I⁢(Y;S,D)=UnqS+UnqD+Shd+Syn,

where UnqS is the unique information that the somatic input conveys about the AP count, UnqD is the unique information that the dendritic input conveys about the AP count, Shd is shared information that both the somatic and dendritic input information possess about the AP count, and Syn is the synergy – the information that the somatic and dendritic inputs, considered together, have about the AP count that cannot be obtained by observing these inputs separately. An advantage of using Williams and Beers’s method for estimating synergy is that it is possible to obtain separate estimates of shared information (redundancy) and synergy, whereas this was not possible in earlier information-theoretic work (Gawne and Richmond, 1993; Schneidman et al., 2003) in which estimates of synergy and shared information, although useful, were conflated (Ince, 2017). Further detail is provided in Supplementary Information.

Trials for which there were no APs for a treatment condition were omitted from consideration. Care was taken to ensure that the input distributions for the treatment conditions considered within a neuron contained exactly the same combinations of somatic and dendritic amplitude. This is particularly important since there is interest in comparing the PIDs obtained under different treatment conditions within each neuron. Ensuring that the input (*S*, *D*) distributions match ensures that any observed difference in a PID component within a neuron is not simply due to a difference in the input distributions.

Data of time-varying input currents and resulting AP times were binned into non-overlapping segments of 120 ms to maximize the joint mutual information (Supplementary Figure 1). Within each bin the AP number, the mean somatic and mean dendritic signals were computed. The values of each of the input signals were binned into quartiles to maximize entropy (Timme and Lapish, 2018). The output was categorized as 0, 1 or 2+ APs. Thus, we generated a 4 × 4 × 3 probability distribution for each of the neurons considered under each of the treatment conditions. PID analyses using the Ibroja method (Bertschinger et al., 2014; Griffith and Koch, 2014), as implemented in the compute UI package (Banerjee et al., 2018), provided values of the partial information coefficients for each distribution. For the purpose of statistical analysis, each of the coefficients was normalized by the joint mutual information *I*(*Y*; *S*, *D*) in each case, so that we analyze their relative contributions to this joint mutual information.

It is well known that measures of mutual information can be biased (Ince et al., 2009). Since the partial information coefficients are defined in terms of mutual information they too are likely to be biased (Timme and Lapish, 2018). Therefore, bias correction was applied using the Delete-1 Jackknife (Efron and Tibshirani, 1993). Further detail is provided in Supplementary Information.

#### Spike Rate Model

The frequency-input (F-I)-curve of somatic current injections was well fitted by a square root function (Supplementary Figures 2A,B; $F=g\,a\,i\,n*\sqrt{I-I_{\text{thr}}}$, for all *I* > *I*_*thr*_). To predict the effect of additional current injection into the apical dendrite, we extended this spike rate model to include a scaled contribution of the dendritic current:

(1) $$F=g\,a\,i\,n*\sqrt{I_{\text{soma}}+D*I_{\text{dend}}-I_{\text{thr}}}$$

for all *I* > *I*_*thr*_; where *I*_*thr*_ denotes the somatic current threshold, *gain* determines the slope, i.e., the overall gain of the input-output function; *I*_*soma*_ and *I*_*dend*_ stand for somatic and dendritic current amplitude, respectively; the dendritic gain factor *D* scales the impact of *I*_*dend*_ relative to *I*_*soma*_. The dendritic current threshold is given by *I*_thr_/*D*. Equation (*1*) was fitted to the data (Figure 2C) to obtain three parameters describing the entire F–I relationship using the Matlab function fminsearch.

#### Regression Analyses

Values for *D* obtained from fits to the experimental F–I data were compared to theoretically predicted values using non-linear regression analysis in GraphPad Prism 6. A regression line was fitted to the scatter plot of predicted versus measured values (Figure 5G). The *Y*-axis intercept was constrained to zero. An extra sum-of-square *F* test was used to test for a significant deviation of the fitted slope from 1 indicating that theoretically predicted values systematically deviated from values derived from fits. Similarly, exponential functions were fitted to scatter plots of normalized input (transfer) resistances versus membrane potential or current step amplitude. Extra sum-of-square F tests were used to statistically compare growth rates between different data sets.

#### Two-Compartment Model

Two compartment models were simulated in the Python-based simulation environment Brian (Goodman and Brette, 2009). Membrane resistances of dendritic and somatic compartment as well as the resistance of the connecting resistor for linear models were derived from electrophysiological measurements of somatic *R*_*in*_, dendritic input resistance (*R*_*dend*_) and transfer resistance *R*_*d,s*_ according to the following formulas. Dendritic membrane resistance:

(2) $$\,R_{\text{md}}=\left(R_{\text{in}}*R_{\text{dend}}-R_{d,s}^{2}\right)/\left(R_{\text{in}}-R_{d,s}\right)$$

(3) $$\mathrm{Axial}\,\mathrm{resistance}:R_{\text{a}}=R_{\text{md}}*\left(R_{\text{in}}-R_{d,s}\,\right)/R_{d,s}$$

(4) $$\mathrm{Somatic}\,\mathrm{membrane}\,\mathrm{resistance}:R_{\text{ms}}=\left(R_{\text{a}}*R_{d,s}\right)/\left(R_{\text{dend}}-R_{d,s}\right)$$

The capacitance for each compartment was estimated from the exponential fit to the decaying phase of small positive current steps at the soma and dendrite, respectively.

To model the voltage-dependence of the transfer resistance, persistent sodium channels were included in the somatic compartment, and HCN (hyperpolarization-activated cyclic nucleotide-gated cation) channels mediating *I*_*H*_ were included in both compartments. Persistent sodium current was modeled as:

(5) $$I_{\text{NaP}}=\left(E_{\text{Na}}-v\right)*g_{{\text{NaP}}_{\text{max}}}*e^{-\frac{\theta_{\text{NaP}}-v}{\sigma_{\text{NaP}}}}$$

θ_*NaP*_ was set to −57.9 mV, and σ_*NaP*_ was set to 6.4 mV (Amarillo et al., 2014).

*I*_*H*_ was modeled as:

(6) $$I_{\text{H}}=\left(E_{\text{H}}-v\right)*g_{\text{H}}$$

(7) $$d\,g_{\text{H}}=\left(g_{\text{H}\,\infty }-g_{\text{H}}\right)/\tau_{\text{H}}$$

(8) $$g_{\text{H}\,\infty }=g_{\text{Hmax}}/\left(1+e^{-\frac{\theta_{\text{H}}-v}{\sigma_{\text{H}}}}\right)$$

θ_*H*_ was set to −80 mV, σ_*H*_ to 12 mV, and τ_*H*_ to 40 ms, similar to published values in the literature (Solomon and Nerbonne, 1993; Berger et al., 2001). The density of HCN and persistent sodium channels was derived from fits of experimentally observed steady-state membrane potential in soma and dendrites in response to somatic and dendritic current steps. The fitting procedure minimized the deviation of the model’s steady state voltage responses to the applied current steps from the experimentally observed steady-state membrane potential responses by adjusting six conductance densities: the somatic and the dendritic leak membrane conductance, the axial conductance (1/*R*_*a*_), somatic and dendritic *g*_*Hmax*_ and somatic *g*_*NaPmax*_. Initial values for the first three parameters were the inverse of *R*_*ms*_, *R*_*md*_, and *R*_*a*_ calculated for the linear model (Eqs. 2–4). Initial values for dendritic and somatic *g*_*Hmax*_ and somatic *g*_*NaPmax*_ were set to 1/*R*_*md*_, 1/20^∗^*R*_*ms*_, and 1/5^∗^*R*_*ms*_, respectively. Minimization was performed by the function minimize from the scipy.optimize package using the Sequential Least Squares Programming optimization algorithm (SLSQP).

#### Multicompartmental NEURON Model

We based our model including most mod files on the model published by Hay et al. (2013). Using the morphology of one of our own neurons, we adjusted the active voltage-dependent conductances according to the algorithm published in the same paper (Hay et al., 2013). We adjusted the apical HCN channel density to values determined during dendritic cell-attached recordings by Kole et al. (2006). To simulate GIRK channel activation, we included a mod file based on the inward rectifier potassium (Kir) channel by Yim et al. (2015). The slope was the only factor that was adjusted to −26.5 mV. This value was based on fits of a sigmoidal voltage-dependent conductance to GIRK currents measured in CA3 pyramidal neurons by Gähwiler and Brown (1985) (Supplementary Figure 3):

(9) $$I\,\left(v\right)=S\,c\,a\,l\,e\cdot \left(v-E_{\text{K}}\right)\cdot \left(g_{\text{min}}+\frac{g_{\text{max}}-g_{\text{min}}}{1+e^{\left(V_{50}-v\right)/S\,l\,o\,p\,e}}\right)$$

with *v* representing the membrane potential, *E*_*K*_ the potassium reversal potential, *g*_*min*_ and *g*_*max*_ the minimal and maximal conductance, *V*_50_ the membrane potential at half-maximal voltage-dependent conductance increase, and the Slope determining the steepness of the sigmoidal. For the fit, all parameters were constrained (i.e., *V*_50_ = *E*_*K*_ = −74.5 mV; *g*_*min*_ = 0; *g*_*max*_ = 1) except for the Scale factor and Slope. The baclofen puff was simulated by inducing a constant time-invariant conductance of 0.135 mS/cm^2^ in the 12 compartments estimated to be directly affected by the baclofen puff (Figure 5F). This resulted in a local membrane hyperpolarization of −1.8 mV in the dendrite and −0.6 mV in the soma comparable to experimentally observed effects (dendrite: −1.6 ± 0.2 mV, soma: −0.7 ± 0.1 mV, *n* = 15).

#### Experimental Design and Statistical Analysis

No statistical methods were used to predetermine sample sizes. The sample size was based on our experience with the high reproducibility of similar experiments (Palmer et al., 2012). Animals of both sexes were used, as we did not observe any obvious sex-specific differences. Experiments and analysis were not conducted blind.

Statistical analyses were performed in GraphPad Prism 6. All pharmacological tests were within experiment comparisons, i.e., the baclofen-induced effect in the presence of nimodipine/tertiapin were compared with the baclofen-induced effect alone in the same cell. Data sets were analyzed with the non-parametric Wilcoxon Signed Rank and the Mann–Whitney tests for paired and unpaired data, respectively. The *p*-values were adjusted using the Bonferroni method, using a familywise error rate of 0.05. The effects of nimodipine/tertiapin on the relative contributions of synergy to the joint mutual information and the baclofen-induced AP frequency reduction for somatic current levels of 250 to 1,000 pA was analyzed using a two-way repeated measures ANOVA. Unless stated otherwise, all data are reported as mean ± s.e.m. The number (n) of observations indicated reflects the number cells recorded from, i.e., biological replicates.

## Results

**FIGURE 1:**
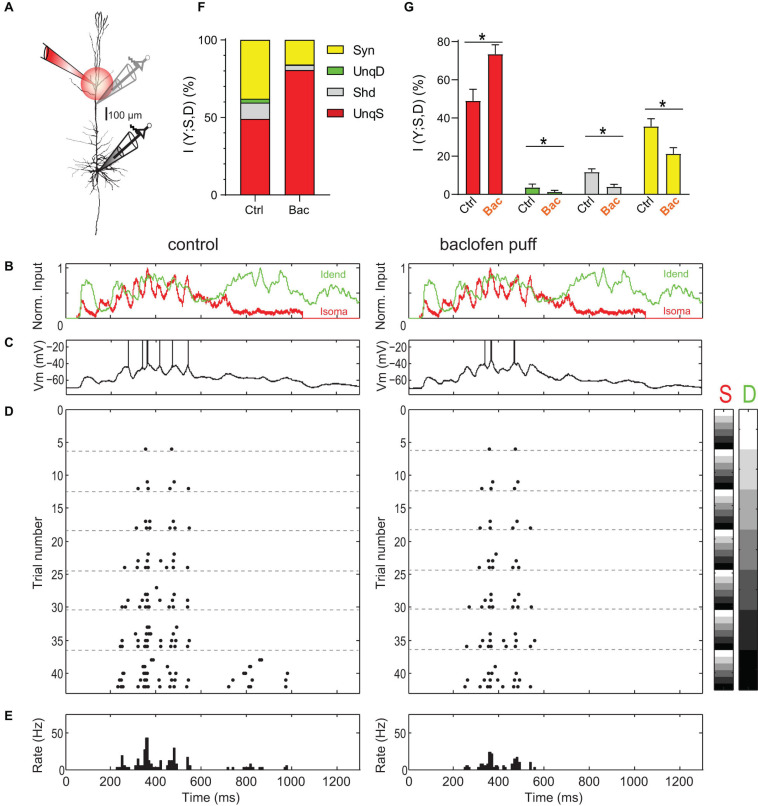
Amplification of somatic AP output by apical dendritic input is inhibited by activation of dendritic GABA_*B*_Rs. **(A)** Left, locations of dual dendritic and somatic patch-clamp recordings are indicated on a biocytin-filled L5 pyramidal neuron. The distance between dendritic and somatic patch electrode was 525 μm. **(B)** Injected current waveforms based on *in vivo* responses to sensory stimulation (Palmer et al., 2012). Dendritic current is shown in green, somatic in red. **(C)** Example membrane potential responses to combined current injections into soma and dendrite in control (left) and during the puff of baclofen (right). Peak current amplitude was 1,000 pA for dendritic and somatic current injections. APs have been clipped. **(D)** Raster plot of APs emitted in individual episodes during increasing levels of dendritic and somatic stimulation strength. Control is shown on the left; raster plot of APs emitted in the same neuron during activation of dendritic GABA_*B*_Rs by a puff of baclofen onto the apical dendrite is shown on the right. Different levels of the injected current in 42 combinations are indicated by the right color bars (S, somatic; D, dendritic). The peak amplitude of the current waveform was increased from 0 pA (white) to 1,250 and 1,500 pA (black) in soma and dendrite, respectively. Step size was 250 pA. **(E)** Peri-stimulus time histogram of APs across all current combinations for both conditions. **(F)** Partial information decomposition (PID) spectra for control condition and during activation of dendritic GABA_*B*_Rs. The contribution of individual PID components is shown as percentage of the total joint mutual information *I*(*Y*; *S*, *D*). Shd, shared information; Syn, synergy; UnqD, unique dendritic information; UnqS, unique somatic information. **(G)** Altered contributions of PID components during dendritic GABA_*B*_R activation. *While UnqS was significantly increased during dendritic GABA_*B*_R activation (adjusted *P* < 0.001, Wilcoxon signed rank test corrected for multiple comparisons using Bonferroni’s method, *n* = 16), UnqD (*P* = 0.0033), Shd (*P* < 0.001) and Syn (*P* < 0.001) were significantly decreased.

We systematically mapped the relationship between AP output to input currents during combined current injections into the soma and apical dendrite of thick-tufted L5b pyramidal neurons in rat (P28–40) somatosensory cortex using dual patch-clamp recordings. The initial resting membrane potential at the dendrite was with −60.7 ± 1.0 mV slightly more depolarized than at the soma (−63.0 ± 0.5 mV, *n* = 25). In a first set of experiments, we used current waveforms to mimic synaptic responses to contralateral hind limb stimulation *in vivo* (Palmer et al., 2012). AP trains were recorded for ≥25 (range: 25–49) combinations of different current levels (Figure 1). We then applied partial information decomposition to assess the relative contributions of information present in the dendritic and somatic input signal about the AP output of the neuron (see section “Materials and Methods”). The joint mutual information *I*(*Y*; *S*, *D*) between the input signals (*S*, somatic; *D*, dendritic) and output (*Y*) was on average 0.80 ± 0.03 bits (*n* = 16 neurons). Of this, information unique to the somatic input signal (UnqS) contributed 49.0 ± 6.0%, while unique dendritic information (UnqD) only contributed 3.7 ± 1.7%. Synergy, i.e., information that the joint variable (*S*, *D*) has about *Y* that cannot be obtained by observing *S* and *D* separately, made up 35.6 ± 4.0% of *I*(*Y*; *S*, *D*). The remainder was shared information that both *S* and *D* have about *Y*. Importantly, our observation of a substantial contribution of synergy, together with a much larger UnqS than UnqD provides strong direct support for apical amplification in cortical pyramidal neurons.

Next, we tested how apical amplification was affected by activation of dendritic GABA_*B*_Rs induced by a puff of baclofen (50 μM) directly onto the apical dendrite (Figure 1A). Puffed baclofen reduced the AP output while having only a small impact on the somatic membrane potential (Figures 1C–E, left vs. right) as previously shown (Palmer et al., 2012). Dendritic GABA_*B*_R activation significantly decreased synergy (adjusted *p* < 0.001, Wilcoxon signed rank test, *n* = 16; Figures 1F,G), while UnqS dominated the information in the AP output (73.4 ± 4.9% vs. 49.0 ± 6.0%, adjusted *p* < 0.001; Figures 1F,G). This demonstrated that GABA_*B*_R-mediated inhibition shifts the balance toward somatic control of AP output and potently decreases apical amplification.

**FIGURE 2:**
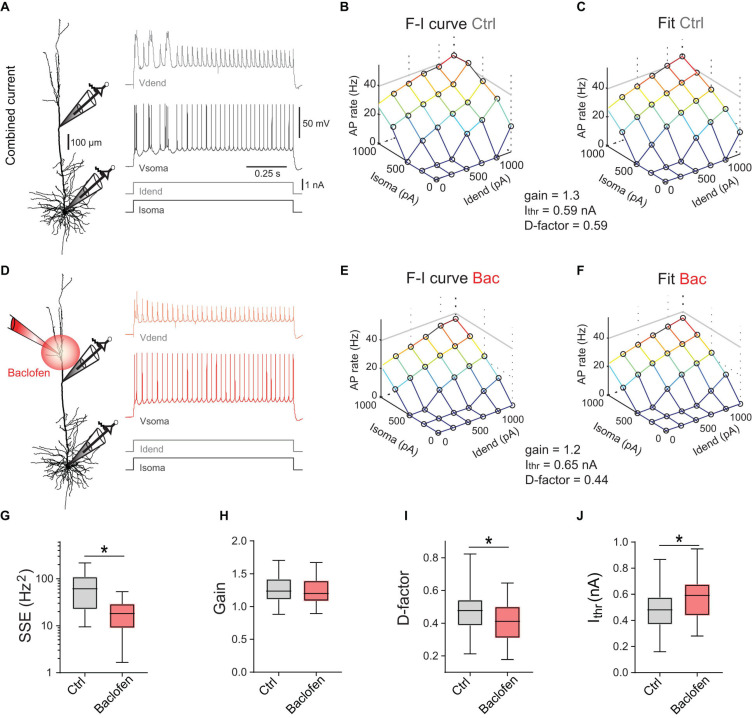
Divisive and subtractive effects of dendritic GABA_*B*_R activation on the F-I relationship. **(A)** Left, locations of dual dendritic and somatic patch-clamp recordings (patch distance = 500 μm) on a L5 pyramidal neuron. Right, dendritic and somatic membrane potential responses to combined current injection. **(B)** F-I relationship for somatic and dendritic current injections. **(C)** Fit of the data to Eq. (1). Parameters from the fit are indicated. **(D)** Membrane potential responses during a puff of baclofen onto the apical dendrite. **(E)** Experimentally observed F-I relationship for the same neuron during dendritic GABA_*B*_R activation. **(F)** Fit of the data to Eq. (1). **(G)** Summed squared errors (SSE) of fit of Eq. (1) to the experimental F-I data. Data during baclofen puff (red) showed significantly lower SSE (*P* < 0.0001, Wilcoxon signed rank test, *n* = 23). **(H)** There was no effect of dendritic GABA_*B*_R activation on the overall gain (in Hz/√pA) of the F-I relationship (*P* = 0.15, Wilcoxon signed rank test, *n* = 25). **(I,J)** Dendritic GABA_*B*_R activation specifically reduced the dendritic gain factor *D* (*P* = 0.0023) and *I*_*thr*_ (*P* < 0.0001). For all grouped data, the entire range of values (whiskers) and the inter-quartile range around the median (boxes) are depicted. Idend, dendritic current; Isoma, somatic current; Vdend, dendritic membrane potential; Vsoma, somatic membrane potential.

How exactly does GABA_*B*_R-mediated inhibition of dendrites reduce apical amplification? We hypothesized that GABA_*B*_R-mediated inhibition may be mainly divisive and change the gain of the input–output function. To investigate the mechanisms underlying the GABA_*B*_R-mediated modulation of apical amplification, we applied constant current steps to soma and dendrite that evoke relatively stable spike rates (Figure 2), which can be more readily interpreted. The frequency-input (F-I)-curve to constant somatic current injections was well fitted by a square root function (Supplementary Figures 2A,B). We extended the F-I-curve to include the effect of additional current injection into the apical dendrite:

(10) $$F=g\,a\,i\,n*\sqrt{I_{\text{soma}}+D*I_{\text{dend}}-I_{\text{thr}}}$$

where *I*_*thr*_ denotes the somatic current threshold; *gain* determines the slope of the input-output function; *I*_*soma*_ and *I*_*dend*_ stand for somatic and dendritic current amplitude, respectively; the dendritic gain factor *D* scales the impact of *I*_*dend*_ relative to *I*_*soma*_. Equation (*1*) was fitted to the data (Figure 2C) to obtain three parameters describing the entire F-I relationship. Next, we measured the F-I relationship during activation of dendritic GABA_*B*_Rs induced by a puff of baclofen (50 μM; Figures 2D,E). Baclofen prevented the activation of dendritic Ca^2+^ spikes and decreased the overall AP output. However, a comparison of the fitted parameters between control condition and baclofen application showed that the overall *gain* was unchanged (*P* = 0.15, Wilcoxon signed rank test, *n* = 25; Figure 2H). In contrast, there was a significant reduction of *D* from 0.48 ± 0.03 to 0.41 ± 0.03 (*P* = 0.0023) demonstrating a divisive effect specifically affecting dendritic inputs (Figure 2I). In addition, there was a significant increase of *I*_*thr*_ from 0.47 ± 0.03 to 0.59 ± 0.04 nA during dendritic GABA_*B*_R activation (*P* < 0.0001; Figure 2J) indicative of an overall subtractive inhibitory effect. These observations show that dendritic GABA_*B*_R-mediated inhibition has both subtractive and divisive components.

### The Role of Ca^2+^ Spikes in Dendro-Somatic Synergy

Inhibition of dendritic L-type Ca^2+^ channels by GABA_*B*_Rs prevents dendritic Ca^2+^ spikes (Perez-Garci et al., 2013) and is therefore expected to strongly reduce the impact of dendritic inputs onto somatic AP output. To test the contribution of Ca^2+^ spikes, we pharmacologically inhibited L-type Ca^2+^ channels by bath application of nimodipine (10 μM; Supplementary Figure 4). However, the reduction of dendritic gain factor *D* from 0.50 ± 0.02 to 0.46 ± 0.02 was surprisingly small (8.2 ± 2.7%, *n* = 7). Baclofen puffed onto the dendrite reduced *D* further by 12.7 ± 5.2% (*P* = 0.047, Wilcoxon signed rank test). An ANOVA of the spike rate reduction after the baclofen puff showed a significant interaction between nimodipine and stimulation intensity [*P* = 0.0166, *F*(3,18) = 4.45] but did not show a main effect of nimodipine [*P* = 0.12, *F*(1,6) = 3.38]. *Post hoc* tests revealed that the presence of nimodipine only moderately decreased the impact of baclofen on the spike rate (Supplementary Figure 4F). This suggested that other mechanisms than direct inhibition of L-type Ca^2+^ channels contributed to the GABA_*B*_R-mediated decrease of the dendritic gain factor *D*.

**FIGURE 3:**
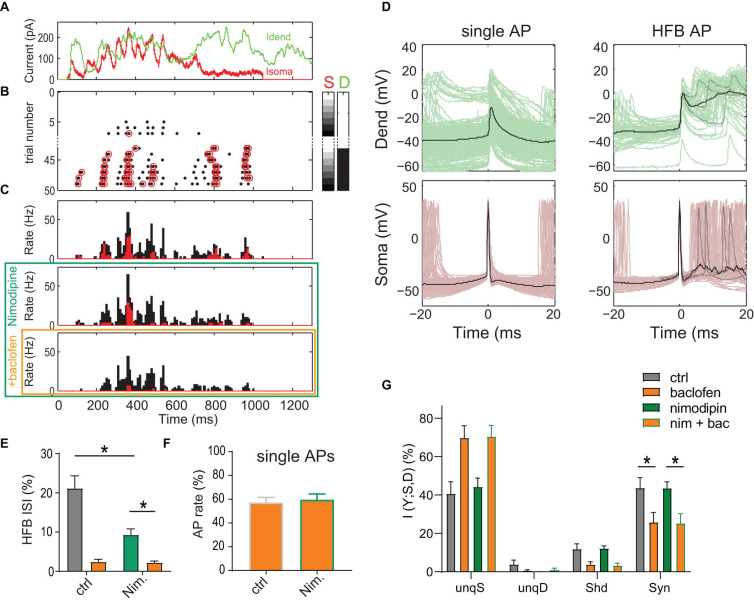
Inhibition of L-type Ca^2+^ channels does not reduce dendro-somatic synergy. **(A)** Injected *in vivo*-like current waveforms. Dendritic current **(D)** is shown in green, somatic (S) in red. **(B)** Raster plot of APs emitted in individual episodes during increasing levels of dendritic and somatic stimulation strength. The seven different levels of the injected current in 49 combinations were applied indicated by the right color bars. Only trials with zero (white) or maximal current in dendrite (peak amplitude = 1,500 pA, black) are shown. Step size was 250 pA. Red circles indicate APs that are part of a HFB (ISI < 15 ms). **(C)** Peri-stimulus time histograms of all (black) and HFB APs (red) for three pharmacological conditions in the same neuron. **(D)** Voltage traces recorded in dendrite and soma for all single APs outside of HFBs (ISI > 15 ms, left) and APs at the start of an HFB (right) in the control condition. Three individual traces (gray) and the mean (black) are highlighted to exemplify the variability of dendritic voltage traces during HFBs. Note the strong association of HFBs with dendritic Ca^2+^ spikes. **(E)** The proportion of HFB ISIs (<15 ms) was significantly decreased by nimodipine (*P* = 0.031; Wilcoxon Signed Rank test, *n* = 6). Baclofen (orange) puffed onto the dendrite further decreased the proportion. **(F)** Normalized AP rate during the dendritic baclofen puff in episodes of low AP probability (<7 Hz, and maximal dendritic current amplitude of 750 pA). The blockade of L-type Ca^2+^ channels by nimodipine did not occlude the effect of baclofen (*P* = 0.73, *n* = 6). **(G)** Relative contributions of PID components in presence and absence of nimodipine with or without a dendritic baclofen puff. Asterisks indicate that synergy was significantly reduced by baclofen under both conditions (adjusted *P* < 0.05; Bonferroni’s multiple comparisons test, *n* = 6).

We proceeded to test the contribution of Ca^2+^ spikes to AP output during dynamic waveform current injections that very reliably evoked dendritic Ca^2+^-spikes (Figures 3A–D). Analysis of the spike-triggered average (STA) of the dendritic and somatic current showed that Ca^2+^ spikes and associated high-frequency bursts of somatic APs (HFBs) were often the result of a synergistic interaction between large dendritic current levels and a rising somatic current (Supplementary Figure 5). When we tested the effect of Ca^2+^ spike inhibition on AP output, we found that nimodipine (10 μM) decreased the relative proportion of HFB ISIs from 21.2 ± 3.2% to 9.3 ± 1.5% of all ISIs (*P* = 0.031; Wilcoxon Signed Rank test, *n* = 6 neurons; Figure 3E). The remaining HFBs were mainly driven by large somatic ramp currents (Supplementary Figure 5C). However, activation of dendritic GABA_*B*_Rs by puffed baclofen further reduced the HFB ISI proportion to 2.2 ± 0.4% comparable to the baclofen-induced effect in the absence of nimodipine (2.4 ± 0.7%). To test whether nimodipine had any effect on the GABA_*B*_R-mediated inhibition in the absence of dendritic Ca^2+^ spikes, we restricted the analysis to episodes with low AP frequency (<7 Hz) and intermediate dendritic current (≤750 pA). Under these conditions, baclofen had the same effect on the AP rate independent of the presence or absence of nimodipine (*P* = 0.73, *n* = 6; Figure 3F). This meant that the reduction of the rate of single APs by dendritic GABA_*B*_Rs was independent of the inhibition of dendritic Ca^2+^ channels.

Most importantly, results from the PID analysis of the entire AP data did not suggest an effect of nimodipine on any information component (Figure 3G). In contrast, there was a main effect of baclofen on synergy [*P* = 0.0021, 2-way repeated-measures ANOVA, *F*(1,5) = 33.65]. *Post hoc* tests revealed that baclofen significantly reduced synergy in the presence of nimodipine (adjusted *P* = 0.0015; Bonferroni’s multiple comparisons test, *t* = 7.285, *n* = 6). These results indicated that inhibition of Ca^2+^ spikes does not reduce apical amplification of AP output, at least on the time scale employed here (bin width of 120 ms; see methods) for the PID analysis of the AP rate.

Taken together, these results demonstrated that direct inhibition of L-type Ca^2+^-channels by dendritic GABA_*B*_Rs plays only a minor role in shaping the overall F-I relationship and strongly suggested that other mechanisms mediate the decrease in the apical amplification of AP output after dendritic GABA_*B*_R activation.

### The Transfer Resistance Is Voltage-Dependent

**FIGURE 4:**
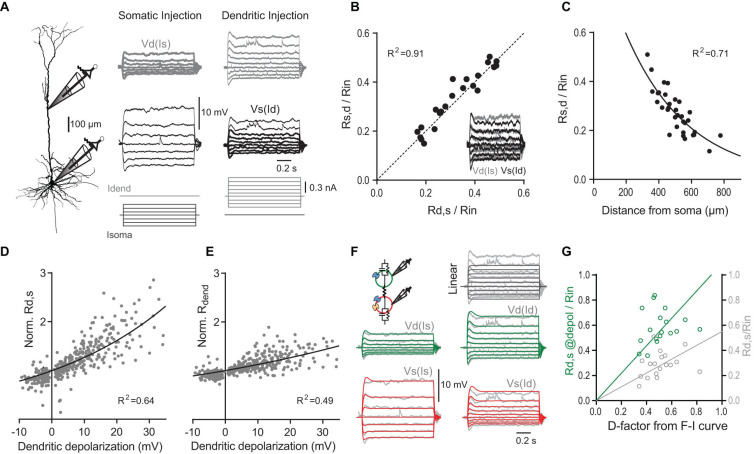
The transfer and dendritic input resistances depend on the membrane potential. **(A)** Left, recording configuration (dendritic to somatic patch distance = 465 μm). Families of current steps were injected into the soma (center) or the dendrite (right). Labels indicate dendritic voltage responses (Vd, gray) to somatic current (Is), and somatic responses (Vs, black) to dendritic current (Id). **(B)** Scatter plot of normalized somato-dendritic transfer resistance (*R*_*s,d*_) versus dendro-somatic transfer resistance (*R*_*d,s*_) show that the transfer resistance is symmetric. Transfer resistances were normalized to *R*_*in*_. Inset shows the overlay of traces from soma (black) and dendrite (gray) for the same family of current injections into the respective opposite compartment (accentuated in **A**). Note that they nicely match indicating symmetry. **(C)** Relationship of normalized *R*_*d,s*_ to distance of dendritic recording location from soma. The mono-exponential fit has a length constant of 383 μm. **(D)** Scatter plot of *R*_*d,s*_ normalized to *R*_*d,s*_ measured at resting membrane potential versus the steady state depolarization at the dendritic recording site for different dendritic current amplitudes (*n* = 38 neurons). Solid line indicates an exponential fit to the data. **(E)** Scatter plot of dendritic input resistance (*R*_*dend*_) normalized to *R*_*dend*_ measured at rest versus the local steady state depolarization. **(F)** A simple two-compartment model was fitted to the recording data from individual neurons. Inset shows a schematic with a dendritic compartment (green) and a somatic compartment (red) connected by a resistor. The model included *I*_*H*_ (blue channels) and persistent sodium (yellow channel) and captured the supralinear voltage-dependence of the recording data. For comparison, voltage responses to dendritic current steps in a linear 2-compartmental model without voltage-dependent conductances are shown in the top right corner. **(G)** Scatter plot of *R*_*d,s*_ normalized to *R*_*in*_ (light gray) versus *D* determined from fits of the F-I data shows a systematic deviation from the identity line [*P* < 0.0001, *F*(1,17) = 94.74]. If *R*_*d,s*_ was measured during large current steps just below AP threshold (@depol; green), the slope is not significantly different from the identity line [*P* = 0.27, *F*(1,17) = 1.31], indicating a close match. Lines were constrained to cross the origin.

Apart from direct amplification of AP output via suprathreshold mechanisms involving dendritic Ca^2+^ spikes, other voltage-dependent mechanisms active at subthreshold membrane potential could potentially mediate the synergistic interaction between dendritic and somatic compartments. An important determinant of the dendritic gain is the transfer resistance from dendrite to the soma (*R*_*d,s*_ = *V*_*soma*_/*I*_*dend*_), which describes the effectiveness of dendritic current in depolarizing the soma (Rall and Rinzel, 1973; Carnevale and Johnston, 1982; Koch, 1984; London et al., 1999; Ulrich and Stricker, 2000). The ratio of the transfer resistance relative to the somatic input resistance (*R*_*d,s*_/*R*_*in*_) determines the dendritic gain factor *D* in Eq. (1) if (*a*) somatic depolarization induced by somatic and dendritic current inputs sum linearly and (*b*) suprathreshold nonlinearities due to dendritic Ca^2+^ spikes are removed. During application of nimodipine, the ratios of *R*_*d,s*_ to *R*_*in*_ (0.28 ± 0.03, *n* = 7) measured near resting membrane potential (see section “Materials and Methods”) were strikingly different to the values of *D* derived from F-I data fits in the presence of nimodipine (0.50 ± 0.02, *P* = 0.016; Wilcoxon signed rank test). This suggested that somatic and dendritic induced depolarizations sum nonlinearly. Therefore, we proceeded to map systematically the current-voltage relationship at subthreshold membrane potentials (Figure 4A).

In linear systems, the transfer resistance is independent of the direction in which it is measured, i.e., it is symmetric (Koch, 1999). In agreement with this prediction, *R*_*d,s*_ closely matched the transfer resistance from soma to dendrite (*R*_*s,d*_) (Figure 4B). As expected, the transfer resistance also depended on the distance between the two recordings sites and decreased with a length constant of 383 μm (Figure 4C). However, larger current steps injected into the dendrite resulted in supralinear increases of the somatic membrane potential response (Figure 4A). Plotting the observed *R*_*d,s*_ normalized by the *R*_*d,s*_ measured at resting membrane potential versus the dendritic membrane potential induced by the current step revealed the strong voltage-dependence of *R*_*d,s*_ (Figure 4D). On average, depolarization of 28.7 mV in the dendrite caused a twofold increase of *R*_*d,s*_. The local dendritic input resistance (*R*_*dend*_) was also modulated by the dendritic membrane potential (Figure 4E); however, this voltage-dependence was with a two-fold increase per 59.5 mV significantly weaker than for *R*_*d,s*_ [*P* < 0.001; extra sum-of-squares *F* test, *F*(3,783) = 90.35].

What are the mechanisms underlying the voltage-dependence of *R*_*d,s*_? A good candidate for mediating the underlying nonlinear conductance are HCN channels, which significantly contribute to the resting conductance in dendrites of pyramidal neurons (Magee, 1998; Williams and Stuart, 2000; Berger et al., 2001; Harnett et al., 2015). The HCN-mediated conductance is highly voltage-dependent with about half of the channels activated at −80 to −95 mV and an *e*-fold current response per 7–10 mV (Solomon and Nerbonne, 1993; Berger et al., 2001). A simple two-compartment model including *I*_*H*_ and persistent Na^+^ channels could reproduce the data (Figure 4F). As expected, the fits showed that there was a high density of *I*_*H*_ specifically in the dendrites (135.4 ± 26.1 nS, versus 52.5 ± 8.8 nS in the soma, *P* = 0.003, *n* = 11 neurons). In contrast, a linear two-compartmental model without any voltage-gated conductances did not capture the observed behavior.

The voltage-dependence of *R*_*d,s*_ could potentially explain why *R*_*d,s*_ normalized by *R*_*in*_ underestimated the dendritic gain factor *D*. Indeed, if *R*_*d,s*_ was measured during large dendritic current injections inducing somatic depolarizations close to the AP threshold, *R*_*d,s*_/*R*_*in*_ provided a much better match of *D* (Figure 4G). Taken together, these results highlighted the synergistic interaction between inputs in the somatic and dendritic compartments even at subthreshold membrane potentials independent of Ca^2+^ channel activation.

### GABA_*B*_R-Activated K^+^ Channels Diminish the Voltage-Dependent Transfer Resistance

**FIGURE 5:**
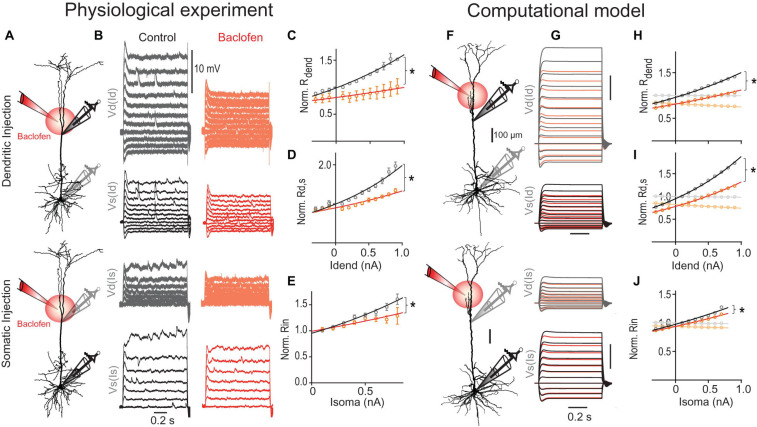
GABA_*B*_R-mediated GIRK channel activation reduces the transfer resistance. **(A)** Schematic of recording arrangement. **(B)** Families of current steps were injected into the dendrite **(top)** or soma **(bottom)** to evoke subthreshold membrane potential responses before (gray) and during the local application of baclofen (red) onto the apical dendrite. Labels indicate dendritic (Vd) and somatic voltage responses (Vs) to dendritic (Id) and somatic current steps (Is). **(C)** *R*_*dend*_ normalized to values at resting membrane potential are plotted versus dendritic current amplitude. Baclofen application significantly increased the doubling interval from 1.4 to 3.3 nA [*P* = 0.0017, *n* = 5 neurons, *F*(1,110) = 10.38] as shown by the exponential fit (solid line). **(D)** Normalized *R*_*d,s*_ versus dendritic current amplitude. Baclofen application significantly increased the doubling interval from 1.0 to 1.8 nA of *R*_*d,s*_ [*P* < 0.0001, *n* = 11, *F*(1,236) = 35.07]. **(E)** Normalized *R*_*in*_ versus somatic current amplitude. Baclofen application significantly increased the doubling interval of *R*_*in*_ from 1.1 to 1.9 nA [*P* = 0.0008, *n* = 11, *F*(1,119) = 11.82]. **(F–J)** A detailed multicompartmental NEURON model containing a realistic HCN channel density but no voltage-gated Ca^2+^ in the dendrites recapitulates the GABA_*B*_R -mediated effect on *R*_*in*_, *R*_*dend*_, and *R*_*d,s*_. The model was based on the morphology **(F)** and recording data from the neuron shown in Figure 4A. **(H)** GIRK channel activation significantly increased doubling interval of *R*_*dend*_ from 1.5 to 2.1 nA [*P* < 0.0001, *F*(1,20) = 115.0]. Traces in lighter colors indicate results from a model without any HCN channels. Note the absence of any voltage-dependent modulation. **(I)** GIRK channel activation increased the doubling interval from 1.0 to 1.3 nA of *R*_*d,s*_ [*P* < 0.0001, *F*(1,20) = 168.4]. **(J)** The voltage-sensitivity of the normalized somatic *R*_*in*_ was significantly decreased by GIRK channel activation [*P* = 0.027, *F*(1,16) = 5.88].

We next tested the effect of dendritic GABA_*B*_R activation on subthreshold membrane potential deflections (Figures 5A,B). When current steps of increasing amplitude were injected into the dendrite, *R*_*dend*_ and *R*_*d,s*_ increased with increasing current step size (Figures 5C,D). Dendritic GABA_*B*_R activation strongly suppressed this voltage-dependent increase of both *R*_*dend*_ [*P* = 0.0017; extra sum-of-squares *F* test, *F*(1,110) = 10.38] and *R*_*d,s*_ [*P* < 0.0001; *F*(1,236) = 35.07]. In addition, current steps of increasing amplitude into the soma induced supralinearly increasing membrane depolarizations in the soma. While dendritic GABA_*B*_R activation had no effect on the somatic *R*_*in*_ measured at resting membrane potential (Figure 5E), it did reduce the nonlinear increase of *R*_*in*_ significantly [*P* = 0.0008; extra sum-of-squares *F* test, *F*(1,119) = 11.82]. These effects may explain why dendritic GABA_*B*_R activation *in vivo* acts as silent inhibition without an apparent effect on the somatic *R*_*in*_ at rest (Palmer et al., 2012).

GABA_*B*_R activation is well known to increase the conductance through GIRK channels (Newberry and Nicoll, 1984). We proceeded to test the effect of GIRK channel activation in a multi-compartmental model including all the major types of voltage-gated ion channels (Figures 5F–J; for details see section “Materials and Methods”). Similar to our recording data, *R*_*d,s*_ of this model showed a stronger voltage-dependent increase than *R*_*dend*_ despite an exponential increase of HCN channel density in distant apical dendrites (Kole et al., 2006). The voltage-dependence of both parameters was completely abolished when the HCN channels were eliminated from the model in agreement with a strong reduction of these parameters by HCN channels at hyperpolarized membrane potentials (Berger et al., 2001). To simulate the effect of the baclofen puff, we included GIRK channels in a fraction of the segments in the apical dendrite estimated to be affected by baclofen in the actual experiment. GIRK channel activation in apical dendrites induced a membrane hyperpolarization of −1.8 and −0.6 mV in apical dendrite and soma, respectively, in close agreement with our experimental observations (dendrite, −1.6 ± 0.2 mV; soma, −0.7 ± 0.2 mV; *n* = 15). This GIRK channel activation was sufficient to suppress the nonlinear increase of both *R*_*dend*_ [*P* < 0.0001; extra sum-of-squares *F* test, *F*(1,20) = 115.0; Figure 5H, orange] and *R*_*d,s*_ [*P* < 0.0001; *F*(1,20) = 168.1; Figure 5I]. Correcting for the small GIRK-induced hyperpolarization by a constant current injection of 70.5 nA into the dendrite was not sufficient to reverse the baclofen-induced effect on *R*_*dend*_ and *R*_*d,s*_ (Supplementary Figure 6), clearly indicating that the GIRK-induced shunt was the primary cause of the decreased effectiveness of dendritic current to depolarize the soma. In the absence of any HCN channels, GIRK channel-induced hyperpolarization was −2.5 and −1.2 mV in apical dendrite and soma, respectively. Under these conditions, the GIRK channel-induced reduction of *R*_*dend*_ (∼20%), *R*_*d,s*_ (∼20%) and the somatic *R*_*in*_ (∼6%) were constant and independent of the membrane potential. Therefore, these results suggest that the interaction of GIRK channel activation with voltage-dependent conductances like HCN channels in the dendrite is a major contributor to reduced voltage-dependence of transfer resistances and hence reduced dendritic gain during dendritic GABA_*B*_R activation.

### Dendritic GIRK Channels Reduce Dendro-Somatic Synergy

**FIGURE 6:**
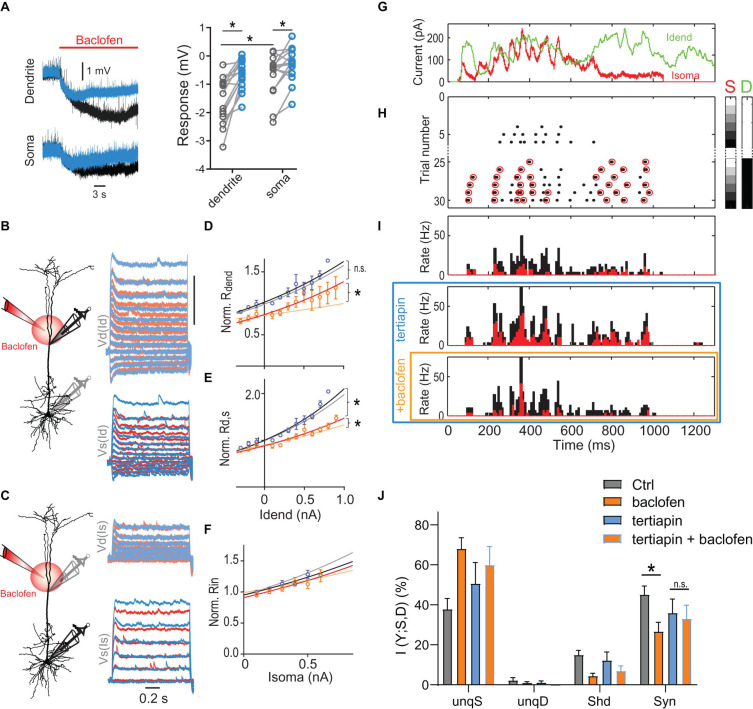
Dendritic GIRK channel-activation reduces dendro-somatic synergy. **(A)** Local hyperpolarization in the dendrite and soma induced by the dendritic baclofen puff and partial block by tertiapin. Significant effects are indicated (*P* < 0.05, *n* = 15, Wilcoxon signed rank test). **(B)** Schematic of recording arrangement. Right, families of current steps were injected into the dendrite to evoke subthreshold membrane potential responses in the presence of tertiapin (0.5 μM, blue) and during dendritic application of baclofen (red). **(C)** The same for somatic current injections. **(D–F)** Tertiapin bath-applied partially blocked GABA_*B*_-mediated effects on input and transfer resistances. Lighter shaded lines indicate the fitted curves in the absence of tertiapin from Figure 5. The voltage-dependence of *R*_*dend*_ and *R*_*d,s*_ was less affected by baclofen in the presence of tertiapin, as indicated by a significantly larger rate constant of the fitted lines [*R*_*dend*_: *P* = 0.033, n1 = 4, n2 = 5, *F*(1,96) = 4.678; *R*_*d,s*_: *P* = 0.0354, n1 = 8, n2 = 11, *F*(1,208) = 4.484]. In addition, the doubling interval of *R*_*dend*_ was no different after baclofen in the presence of tertiapin compared to tertiapin alone [2.2 nA vs. 1.9 nA; *P* = 0.48, *n* = 4 neurons, *F*(1,71) = 0.5063]. **(G)** Injected *in vivo*-like current waveforms. Dendritic current **(D)** is shown in green, somatic (S) in red. **(H)** Raster plot of APs emitted in individual episodes during increasing levels of dendritic and somatic stimulation strength. The six different levels of the injected current in 30 combinations were applied indicated by the right color bars. Only trials with zero (white) or maximal current in dendrite (peak amplitude = 1,000 pA, black) are shown. Step size was 250 pA. Red circles indicate APs that are part of a HFB (ISI < 15 ms). **(I)** Peri-stimulus time histograms of all (black) and HFB APs (red) for three pharmacological conditions in the same neuron. **(J)** Relative contributions of PID components in presence and absence of tertiapin with or without a dendritic baclofen puff. Asterisk indicates that synergy was significantly reduced by baclofen under control conditions (adjusted *P* = 0.0121; Bonferroni’s multiple comparisons test, *t* = 5.310, *n* = 5). The change of synergy in the presence of tertiapin was not significant (n.s., adjusted *P* = 0.31; *t* = 0.0121).

We tested experimentally the contribution of GIRK channels by applying the GIRK antagonist tertiapin (0.5 μM). Tertiapin reduced the baclofen-induced hyperpolarization of −1.6 ± 0.2 mV in the dendrite by 54.1 ± 7.6% (*n* = 15), consistent with a functionally significant contribution of GIRK channels to the baclofen-induced hyperpolarization (Figure 6A). The incomplete block of the baclofen-induced hyperpolarization by tertiapin is in agreement with recent reports suggesting that other K^+^ channels such as two-pore domain K^+^ channels also contribute to GABA_*B*_-mediated membrane potential hyperpolarizations in L5 pyramidal neurons and entorhinal stellate cells (Deng et al., 2009; Breton and Stuart, 2017). Importantly, tertiapin partially blocked the effect of baclofen on the voltage-dependence of *R*_*dend*_ and *R*_*d,s*_ (Figures 6B–F). This observation confirmed the important contribution of GIRK channel activation in the dendrites to the altered integrative properties of L5 pyramidal neurons after dendritic GABA_*B*_R activation. We proceeded to test the effect of GIRK channel activation on AP output.

Bath application of tertiapin increased the somatic *R*_*in*_. This resulted in greater cellular excitability reflected by increased values of the F-I gain (Supplementary Figure 7A). Both observations suggested that there was a baseline activity of GIRK channels in the control condition (Chen and Johnston, 2005). In the presence of tertiapin, puffed baclofen was less effective in reducing AP output during current steps (Supplementary Figures 7B–D). ANOVA analyses of the baclofen-induced AP rate reduction showed that there was a main effect of tertiapin [*P* = 0.0131, *F*(1,6) = 12.14, *n* = 7], as well as a statistically significant interaction between tertiapin and stimulation-intensity [*P* = 0.0083, *F*(3,18) = 5.34]. These results confirmed that dendritic GABA_*B*_R-mediated GIRK channel activation significantly contributed to reduced somatic AP output.

To test directly the effect of GIRK channel activation on dendro-somatic synergy, we applied PID analysis to AP output during dynamic waveform current injections in the presence and absence of tertiapin within the same neurons (Figures 6G–J). This analysis showed that there was a significant interaction between tertiapin and baclofen [*P* = 0.0349; 2-way repeated-measures ANOVA, *F*(1,4) = 9.843] besides a main effect of baclofen on synergy [*P* = 0.0009; *F*(1,4) = 79,17]. *Post hoc* tests revealed that while baclofen significantly reduced synergy under control conditions (adjusted *P* = 0.0121; Bonferroni multiple comparisons test, *t* = 5.310, *n* = 5), the presence of tertiapin prevented this reduction (adjusted *P* = 0.34; *t* = 0.0121; Figure 6J). Together, these results demonstrated that dendritic GIRK channel activation partially mediated the reduction of dendro-somatic synergy.

### Synaptically Activated Dendritic GIRK Channels Control Somatic AP Output

Cortical neurogliaform (NGF) cells are known to reliably activate postsynaptic GABA_*B*_Rs (Tamas et al., 2003; Price et al., 2008). A recent study demonstrated that a large fraction of L1 interneurons in the mouse, which express Neuron-Derived Neurotrophic Factor (NDNF), inhibits postsynaptic pyramidal neurons by a combination of slow GABA_*A*_R and GABA_*B*_R-mediated inhibitory postsynaptic current as it is typical for NGF cells (Abs et al., 2018). However, it is not known whether GIRK channels activated by NDNF^+^ interneurons can effectively reduce somatic AP output in L5 pyramidal neurons.

**FIGURE 7:**
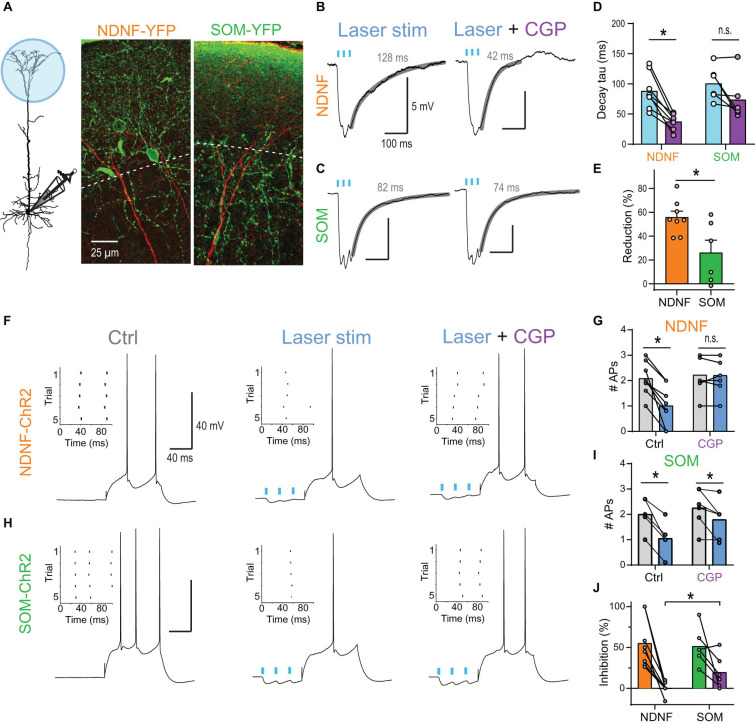
NDNF-positive NGF cells effectively reduce AP output via activation of dendritic GABA_*B*_Rs. **(A)** Left, schematic of recording arrangement during optogenetic stimulation of GABAergic inputs onto the apical tuft in NDNF-ChR2 and SOM-ChR2 mice. Center, immuno-histochemical labeling of YFP (green) in a slice from a NDNF-ChR2 mouse shows the localization of most NDNF-cell bodies and cellular processes within layer 1 (above the dashed line). The apical dendrite of a biocytin-filled L5b pyramidal neuron is shown in red. Right, cellular processes of SOM interneurons were present in both layer 1 and 2 in SOM-ChR2 mice. **(B)** Optogenetic burst stimulation (3@40 Hz, 5 ms pulses of 470 nm laser light) of NDNF interneurons expressing ChR2 evoked IPSPs with a slow decay recorded in the pyramidal cell soma. Fit to decay phase and measured weighted tau are indicated (gray). Block of GABA_*B*_Rs by wash-in of 2 μM CGP54626 strongly reduced the decay suggesting a strong contribution of GABA_*B*_R-activated GIRK channels to the IPSP. **(C)** Example traces of optogenetically evoked burst IPSPs from SOM interneurons before and after application of 2 μM CGP54626. **(D)** Group data of weighted decay taus. Asterisk indicates CGP54626-induced reduction in NDNF-ChR2 IPSPs (*P* = 0.0078; Wilcoxon signed rank test, *n* = 8). **(E)** Relative reduction of the weighted tau shows significantly larger GABA_*B*_R-mediated components after the activation of NDNF vs. SOM interneurons (*P* = 0.029; Mann–Whitney test, n1 = 8, n2 = 6). **(F)** Example traces showing the effect of NDNF-ChR2 on subsequent AP output. Left, a 100 ms current step reliably evoked 2 APs. Inset shows the raster plot for 5 consecutive trials. Center, optogenetic burst stimulation of NDNF interneurons preceding the current step reduced the AP rate. Right, the inhibition was blocked by the addition of the GABA_*B*_R antagonist CGP54626 (2 μM). **(G)** Group data showing the complete block of AP inhibition by CGP (adjusted *P* > 0.99, *n* = 8 neurons, Bonferroni’s multiple comparisons). **(H,I)** The same for optogenetic burst stimulation of SOM interneurons. Asterisks indicate significant effects of the laser (adjusted *P* < 0.05, *n* = 6 neurons, Bonferroni’s multiple comparisons). **(J)** Relative reduction of AP output for both interneuron subtypes show that CGP had a significantly larger effect on NDNF- than on SOM-ChR2-mediated inhibition (*P* = 0.0176; Mann–Whitney test, n1 = 8, n2 = 6).

Optogenetic burst stimulation (3 stimuli @ 40 Hz) of inputs onto the apical tuft of L5 pyramidal neurons in mouse brain slices evoked prominent inhibitory PSPs (IPSPs) with a noticeably slow decay when recorded at a depolarized membrane potential (∼−55 mV; Figures 7A,B). The block of GABA_*B*_Rs by wash-in of 2 μM CGP54626 strongly reduced the decay tau (from 88.2 ± 10.4 to 37.4 ± 4.5 ms, *n* = 8) indicating a strong contribution of GABA_*B*_R-activated GIRK channels to the IPSP (Figures 7B,D). The same burst stimulation presented prior to a somatic current step reduced the AP discharge from 2.1 ± 0.2 to 1.0 ± 0.3 APs (adjusted *P* < 0.001, *n* = 8 neurons, Bonferroni’s multiple comparisons; Figures 7F,G). This effect was entirely GABA_*B*_R-dependent, as the application of 2 μM CGP54626 prevented any AP rate reduction (2.2 ± 0.2 APs vs. 2.2 ± 0.2, *n* = 8).

Next, we tested whether another major class of dendrite-targeting interneurons also activates GABA_*B*_Rs by optogenetic stimulation of somatostatin (SOM) interneurons. Induced burst IPSPs exhibited a slow decay (weighted tau of 100.6 ± 11.4 ms, *n* = 6), but were less sensitive to CGP54626 (73.6 ± 14.9 ms, *n* = 6; Figures 7C–E), indicating a reduced contribution of GABA_*B*_Rs. Indeed, stimulation of SOM inputs prior to a somatic current step continued to reduce AP discharge even after the wash-in of the GABA_*B*_R antagonist CGP54626 (Figures 7H–J). This suggested that the slow component of the IPSP was only partially mediated by GABA_*B*_Rs and that slow GABA_*A*_R-mediated inhibition contributed (Schulz et al., 2018; Zorrilla de San Martin et al., 2020).

Taken together, these results show that synaptic activation of dendritic GIRK by NGF cells provides sufficient inhibition to control AP output in L5 pyramidal neurons.

## Discussion

By systematically mapping the input-output relationship in L5b pyramidal neurons, we demonstrated here for the first time that the voltage-dependence of *R*_*d,s*_ significantly contributes to apical amplification. To demonstrate its impact during physiological relevant input patterns, we applied the unbiased concept of dendro-somatic synergy. Dendritic GABA_*B*_R-activated K^+^ channels greatly reduced the voltage-dependent *R*_*d,s*_ and synergy, while having negligible effects on *R*_*in*_. This explains the dramatic shift of the information flow toward perisomatic feedforward inputs with a UnqS of 75% during GABA_*B*_R activation. It also provides a mechanistic foundation of interhemispheric inhibition of AP output in the absence of a discernable membrane hyperpolarization observed *in vivo* (Palmer et al., 2012). Together, our results stress the bidirectional nature of the nonlinear interaction between dendritic and somatic compartments and highlight the powerful control of dendro-somatic synergy by apical dendritic GABA_*B*_Rs.

### The Significance of the Voltage Dependence of the Transfer Resistance

The contribution of dendritic K^+^ channels to the GABA_*B*_R-mediated inhibition of AP output was much more pronounced than previously thought (Breton and Stuart, 2012; Palmer et al., 2012). The main reason for the powerful impact of the seemingly small hyperpolarization by GABA_*B*_R-activated K^+^ channels was due to its impact on other voltage-dependent conductances in the dendrites. While *R*_*in*_ and *R*_*dend*_ were weakly voltage dependent, *R*_*d,s*_ strongly depended on the dendritic membrane potential with a twofold increase per 28.7 mV. Consequently, the impact of dendritic inputs on the somatic membrane potential grew supralinearly with increasing dendritic membrane potential depolarization.

The voltage-dependent deactivation of *I*_*H*_ is probably the most important factor mediating the nonlinear *R*_*d,s*_. At rest, *I*_*H*_ decreases the impact of small EPSPs evoked in the distal dendrite at the soma (Golding et al., 2005; George et al., 2009) and contributes to increased compartmentalization of synaptic inputs (Harnett et al., 2015). However, larger depolarization deactivates *I*_*H*_ thereby increasing the local input resistance and decreasing the leakage for dendritic current flowing to the somatic compartment. Experimental and computational studies using white-noise and sinusoidal current waveforms have shown that *I*_*H*_ is the main contributor to the resonance in the theta frequency range at 4–10 Hz of pyramidal neuron dendrites, which behave otherwise like low-pass filters (Ulrich, 2002; Das and Narayanan, 2014; Kalmbach et al., 2017). The present study shows that the depolarization-induced deactivation of I_*H*_ dramatically increases the impact of apical dendritic inputs on the soma during sustained dendritic input currents and forms the basis for dendro-somatic synergy.

Two technical limitations of our approach were that we injected currents with defined amplitudes at just two different locations within the complex dendritic tree. Thus, we neglected any supralinear interactions on a finer anatomical scale, namely between individual synaptic inputs and individual dendritic branches (Larkum et al., 2009). In the case of real synaptic conductances, the current depends on the actual membrane potential. Therefore, it is likely that the described voltage-dependent interaction between dendritic and somatic compartment is somewhat weaker for PSCs mediated by voltage-insensitive conductances like AMPA receptors, as the depolarization diminishes the synaptic driving force. However, the voltage-dependent conductance of NMDARs strongly increases with depolarization. NMDARs not only mediate supralinear interactions between multiple synaptic inputs, they will also contribute to a large supralinear increase of the interaction between dendritic and somatic compartments at depolarized membrane potentials. Therefore, dendritic depolarization by glutamatergic synapses is expected to show a similarly strong supralinear effect on the somatic membrane potential under more physiological conditions.

### Mechanisms of GABA_*B*_R-Mediated Modulation of Dendritic Integration

Dendritic GABA_*B*_R activation greatly lowers *R*_*dend*_. Although the membrane hyperpolarization induced by the dendritic puff of baclofen appeared to be small (Breton and Stuart, 2012; Palmer et al., 2012), the strong impact on *R*_*dend*_ suggests that the conductance change was much larger. This apparent mismatch is caused by active HCN channels that counteract any hyperpolarization induced by GIRK channels and contribute to the shunt themselves. Interestingly, HCN channels are structurally and functionally associated to GABA_*B*_Rs (Schwenk et al., 2016). The combined effect of GIRK and HCN channel activation decreased *R*_*d,s*_ and consequently the impact of dendritic inputs on somatic AP discharge.

Interestingly, direct inhibition of Ca^2+^ spikes did not affect dendro-somatic synergy in our PID analysis. We assessed synergy on the level of the AP rate; however, neuronal computations in the brain are probably based on a combination of rate code and precise AP timing code, where short HFBs may be of fundamental importance (Lisman, 1997; Naud and Sprekeler, 2018; Doron et al., 2020). Therefore, dendritic Ca^2+^ spikes are expected to contribute to synergy on a finer time scale. The technical limitations of the PID analysis did not allow for a statistical evaluation of this effect on the current data set (see section “Materials and Methods”). The contribution of Ca^2+^ spikes to dendro-somatic synergy will have to be tested in the future using input signals that are more strongly modulated over time in combination with improved information analysis tools that also take the dynamics of the neuronal response into account (Selimkhanov et al., 2014). Dendritic GABA_*B*_R activation is expected to minimize dendro-somatic synergy under these conditions nevertheless due to direct inhibition of L-type Ca^2+^ channels.

### Neurogliaform Cells Are Responsible for Dendritic GABA_*B*_R Activation

GABA_*B*_Rs are thought to be activated by spill-over of GABA from synaptic release sites (Scanziani, 2000; Kohl and Paulsen, 2010). In principle, any dendrite targeting interneuron population could activate dendritic GABA_*B*_Rs in L5 pyramidal neurons given that they release sufficient amounts of GABA to overcome the effective perisynaptic GABA reuptake mechanisms (Destexhe and Sejnowski, 1995; Thomson and Destexhe, 1999). Thus, tonic activity of SOM interneurons has been shown to downmodulate glutamate release via presynaptic GABA_*B*_Rs (Urban-Ciecko et al., 2015). However, our results indicate that NGF cells may be of particular relevance for the modulation of dendritic properties via postsynaptic GABA_*B*_Rs. While activation of both NDNF and SOM interneurons resulted in slow inhibition that was sufficient to reduce somatic AP firing in pyramidal neurons in the mouse, only NDNF interneuron-mediated inhibition was highly sensitive to a GABA_*B*_R antagonist. This is in agreement with previous reports of reliable GABA_*B*_R activation after single presynaptic APs in NGF cells recorded in the rat (Tamas et al., 2003; Price et al., 2008; Jiang et al., 2013). These observations suggest that, while the precise size of the postsynaptic response in the soma may vary between species depending on the electrotonic structure of the pyramidal neuron, the fundamental connectivity motive of presynaptic interneuron to postsynaptic neuron and receptor type is evolutionarily conserved.

Two anatomical features may contribute to the efficient GABA_*B*_Rs activation: the exceptional high density of about 1 bouton per 2.5 μm axon and the greater than usual distance of boutons from their target dendrites (Olah et al., 2009; Overstreet-Wadiche and McBain, 2015). Hence, GABA released by NGF cells is thought to act via volume transmission potentially affecting many postsynaptic targets simultaneously rather than by ‘point-to-point’ synaptic transmission. Therefore, NGF cells in the superficial cortical layers are the prime candidates to activate dendritic GABA_*B*_Rs in L5 pyramidal neurons under most conditions.

### Functional Significance of GABA_*B*_Rs Activation in Dendrites

**FIGURE 8:**
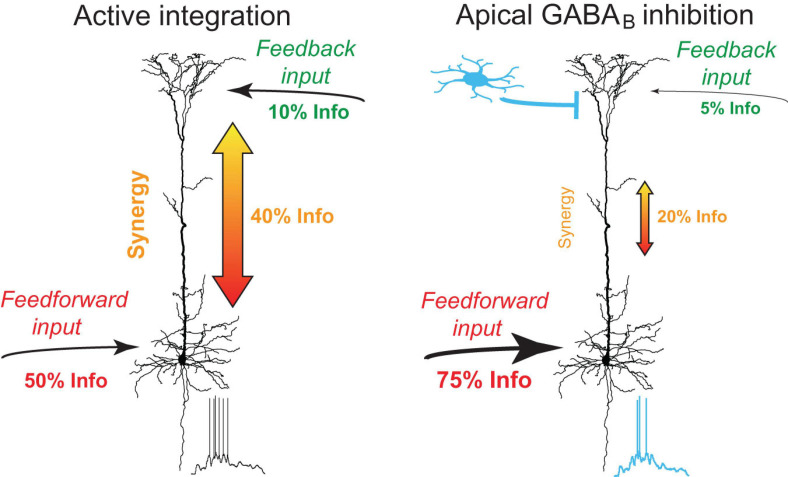
Summary of dendro-somatic synergy and its regulation by apical GABA_*B*_R activation. In the absence of inhibition, feedback inputs from higher-order areas onto the apical dendrites of L5 pyramidal neurons synergistically amplify the effect of feedforward inputs on AP output. Dendro-somatic synergy makes up ∼40% of joint mutual information between synaptic input signals and AP output. Right, during activation of apical GABA_*B*_Rs by interneurons like NGF cells, the influence of apical dendritic inputs and dendro-somatic synergy is halved due to the impact of dendritic potassium channels. The somatic input signal now dominates with ∼75% the information content of the AP output.

Neurogliaform cells receive inputs from many afferent cortical and subcortical areas including higher-order thalamic and cortical areas (Craig and McBain, 2014; Abs et al., 2018; Pardi et al., 2020; Anastasiades et al., 2021). The sensory recruitment of NGF cells is profoundly enhanced after the association of a sensory cue with a high behavioral salience (Abs et al., 2018). Our results suggest that GABA_*B*_R activation by NGF cells down-regulates apical amplification in a large population of neighboring pyramidal neurons and shifts the information flow toward perisomatic feedforward inputs. While synaptic recruitment of NGF cells is likely associated with stronger excitatory drive to the dendritic tuft of neighboring L5 pyramidal neurons, the very slow dynamics of metabotropic inhibition suggest that the inhibitory effect considerably outlasts the direct excitatory effects (Kohl and Paulsen, 2010; Palmer et al., 2012). In the absence of GABA_*B*_R -mediated inhibition, feedback inputs from higher-order areas onto the apical dendrites of L5 pyramidal neurons synergistically amplify the effect of feedforward inputs on AP output due to the voltage dependence of R_*d,s*_, a process that is probably fundamentally important in active sensing (Xu et al., 2012; Takahashi et al., 2016). Together, these observations suggest that NGF cells are at a unique position to gate information flow of feedback inputs and to shift information processing from a top-down mode toward a feedforward bottom-up mode (Figure 8).

## Conclusion

This study demonstrates that the voltage-dependent *R*_*d,s*_ forms the basis for dendro-somatic synergy in L5 pyramidal neurons. Distal dendritic GABA_*B*_Rs control the non-linear integration of dendro-somatic inputs via a GIRK channel-mediated shunt. This novel inhibitory mechanism is likely to be an important regulator of information flow in the cortex.

## Data Availability Statement

The data supporting the conclusions of this article will be made available by the authors to any qualified researcher.

## Ethics Statement

The animal study was reviewed and approved by Veterinärdienst, Amt für Landwirtschaft und Natur des Kantons Bern; Veterinäramt, Gesundheitsdepartement des Kantons Basel-Stadt.

## Author Contributions

JMS and MEL designed the study. JMS collected all data and wrote the manuscript. JMS and JWK analyzed the data. JB and MEL acquired the necessary funding and supervised the study. All authors reviewed and edited the manuscript and approved the submitted version.

## Conflict of Interest

The authors declare that the research was conducted in the absence of any commercial or financial relationships that could be construed as a potential conflict of interest.

## Publisher’s Note

All claims expressed in this article are solely those of the authors and do not necessarily represent those of their affiliated organizations, or those of the publisher, the editors and the reviewers. Any product that may be evaluated in this article, or claim that may be made by its manufacturer, is not guaranteed or endorsed by the publisher.

## Footnotes

1
https://imagej.net/

## Supplementary Material

Supplementary Figure 1 Dependence of joint mutual information on bin size of the PID analysis.

Supplementary Figure 2 Extension of the spike rate model for dendritic current injections.

Supplementary Figure 3 GIRK-mediated currents measured in CA3 pyramidal neurons from Figure 4B in Gähwiler and Brown (1985).

Supplementary Figure 4 GABA_*B*_R-mediated inhibition of L-type Ca^2+^ channels prevents burst firing.

Supplementary Figure 5 Differential regulation of single APs and HFBs during *in vivo*-like input waveforms by dendritic GABA_*B*_Rs.

Supplementary Figure 6 The conductance change induced by dendritic GIRK channels reduces the transfer resistance.

Supplementary Figure 7 GIRK channel-activation in dendrites decreases action potential output.

Supplementary Information Supplementary notes on partial information decomposition.
