# Supplementary material for: GABAB Receptor-Mediated Regulation of Dendro-Somatic Synergy in Layer 5 Pyramidal Neurons
Source: Front Cell Neurosci. 2021 Aug 25;15:718413. doi: 10.3389/fncel.2021.718413 (PMC8425515; doi:10.3389/fncel.2021.718413)
Supplement: Supplementary Figure 1 — Dependence of joint mutual information on bin size of the PID analysis. [file Presentation_1.pdf]

SUPPLEMENTARY INFORMATION

1 Supplementary Notes on Partial Information Decomposition

Williams and Beer's definition [1] of the partial information decomposition (PID) involves three classical measures of mutual information [2]: $I(Y;S)$ the information shared by the somatic input, S and the action potential (AP) output, Y, $I(Y;D)$ the information shared between the dendritic input, D and the AP output, as well as $I(Y;S,D)$ the information shared between the inputs, (S,D), considered jointly, and the output, Y – the *joint mutual information* (JMI). The defining equations of the four partial information components are:

$$I(Y;S) = \text{Unq}S + \text{Shd} \tag{1}$$

$$I(Y;D) = \text{Unq}D + \text{Shd} \tag{2}$$

$$I(Y;S,D) = \text{Unq}S + \text{Unq}D + \text{Shd} + \text{Syn}. \tag{3}$$

The partial information decomposition has been applied to data in neuroscience; see, e.g. [3, 4, 5, 6, 7]. For a recent overview, see [8]. For further discussion of the role of contextual guidance in a neuronal modulatory interaction and also apical amplification, see [9, 10]. Some further useful equations are

$$I(Y;S,D) = I(Y;S) + I(Y;D|S) = I(Y;D) + I(Y;S|D), \tag{4}$$

$$I(Y;S|D) = \text{Unq}S + \text{Syn}, \tag{5}$$

$$I(Y;D|S) = \text{Unq}D + \text{Syn}, \tag{6}$$

$$I(Y;S) - I(Y;D) = \text{Unq}S - \text{Unq}D \tag{7}$$

A particularly important use of the PID is to separate synergy from shared information in a probabilistic system. This is not possible using classical measures of mutual information; only lower bounds can be obtained. For example, the interaction information [11], when non-negative, defined by

$$II(Y;S;D) = I(Y;S,D) - I(Y;S) - I(Y;D) = \text{Syn} - \text{Shd},$$

was used [12, 13] to estimate synergy, while the negative of the interaction information, termed 'coinformation', when non-negative, was used [14] to extract shared information in a contextually-guided artificial neural network.

The conditional mutual information, $I(Y;D|S)$, defined in [2], is a measure of the conditional dependence between the dendritic input and the AP count, having taken the somatic input into account. The mutual information $I(Y;S)$ is a measure of the marginal dependence between the AP count and the somatic input. It is useful to consider such classical measures of information. We consider them in Table 1 for the particular neuron considered in Fig. 1E.

Table 1: **Information measures for the neuron in Fig. 1E**.

Some classical information measures (the unit is bit), given to three significant figures and also as percentages of the joint mutual information

Baclofen	$I(Y;S)$	$I(Y;D)$	$I(Y;S\|D)$	$I(Y;D\|S)$	$I(Y;S,D)$	$II(Y;S;D)$
Absent	0.332	0.072	0.486	0.226	0.558	0.154
	(59.5)	(13.0)	(87.0)	(40.5)	(100)	(27.5)
Present	0.433	0.018	0.498	0.083	0.516	0.065
	(83.9)	(3.6)	(96.4)	(16.1)	(100)	(12.5)

Considering marginal distributions we notice that, since $I(Y;S)$ is much larger than $I(Y;D)$, the AP count is much more strongly related to the somatic input than the dendritic input when baclofen is absent, and more strongly so when baclofen is present. From (7), we may deduce, without computing a PID, that the unique information transmitted about the somatic input is much larger than that transmitted about the dendritic input. The measure of conditional dependence between the somatic input and the AP count, $I(Y;S|D)$, is a large percentage of the JMI when baclofen is absent, and a higher percentage when baclofen is present. Of more interest to contextual modulation and apical amplification are the measures of conditional dependence between the dendritic input and the AP count, $I(Y;D|S)$. This is 40.5% of the JMI when baclofen is absent, but 16.1% in the presence of baclofen. The difficulty with this classical measure in respect of consideration of apical amplification is that, from (6), this measure is composed of synergy and a component unique to the dendritic input. Therefore, given only the value of $I(Y;D|S)$ one cannot know whether it indicates all synergy or all unique information, or a mixture of the two. Partial information decomposition makes this distinction possible.

From the measures of interaction information we can conclude, without computing a PID, that in the absence of baclofen at least 27.5% of the JMI is due to synergy, whereas when baclofen is present at least 12.5% of the JMI is due to synergy.

We note also that since the values of the JMI are different under the two baclofen conditions we consider the partial information components as percentages of the joint mutual information under each condition. They are given in Table 2.

Table 2: **Partial information decompositions for the neuron in Fig. 1E**.

Partial information components given as a percentage of the joint mutual information.

Baclofen	Unq$S$	Unq$D$	Shd	Syn
Absent	49.0	2.5	10.5	38.0
Present	80.5	0.2	3.4	15.9

Whether baclofen is absent or present there is evidence for apical amplification for the following reasons: there is an appreciable amount of synergy; the unique information due to the somatic input is much larger than that from the dendritic input, suggesting that the somatic input is the primary driver in the information transmission, with the dendritic input in a secondary, modulatory role; the unique information due to the dendritic inout is very small.

When baclofen is absent, the somatic and dendritic inputs combine to transfer 38% of the joint

mutual information as synergy, and they contribute to transferring 10.5% as shared information. The unique information transmitted about the somatic input is large, at 49% of the joint mutual information, but the unique information about the dendritic input is only 2.5%. This indicates that the dendritic input can have a large effect on the AP count via the synergistic component while conveying very little information about itself.

When baclofen is present, the somatic and dendritic inputs combine to transfer 15.9% of the joint mutual information as synergy, and they contribute to transferring 3.4% as shared information. The unique information transmitted about the somatic input is verylarge, at 80.5% of the joint mutual information, but the unique information about the dendritic input is only 0.2%. This indicates that the dendritic input can have a large effect on the AP count via the synergistic component while conveying very little information about itself.

Due to the reduction in the values of synergy and shared information, and the consequent increase in unique information due to the somatic input, it is clear for this neuron that there is less apical amplification in the presence of baclofen.

2 Bias correction

It is well-known that classical measures of mutual information are biased upwards and various efforts have been made to correct such biases [15, 16]. Since partial information components are derived from mutual informations it is expected that they too are biased. Here, the Delete-1 Jackknife [17] has been implemented to correct biases in the classical information measures as well as the partial information components. Since this method deals linearly with the statistics involved, it has the important property that the bias-corrected versions of the partial information components, as well as the mutual informations, also satisfy Equations (1) - (3). The jackknife and other resampling methods have been applied in neuroscience [18, 19].

The method is straightforward. When the data on (Y, S, D) is binned into frequencies, prior to forming probability distribution, suppose that the total frequency is N, which means that there are N observations. Each term in Equations (1) - (3) is estimated using the given probability distribution. Let θ be one of the terms. We have an estimate, $\hat{\theta}$, of this term based on the entire dataset of N observations. Then one observation is deleted in turn to give N datasets (jackknife samples), each of which contains $N - 1$ observations. Then each term in Equations (1) - (3) is estimated using each of these N datasets, and the mean values found, $\hat{\theta}_{(.)}$ for our generic term. The bias-corrected estimate of θ is

$$\hat{\theta}_{bc} = N\hat{\theta} - (N-1)\hat{\theta}_{(.)}.$$

References

[1] Williams PL, Beer RD. Nonnegative decomposition of multivariate information. arXiv 2010, arXiv:1004.2515. Available online: https://arxiv.org/abs/1004.2515 (accessed on 20 February 2019).

[2] Cover TM, Thomas JA. Elements of Information Theory. New York, USA: Wiley-Interscience; 1991.

[3] Wibral M, Finn C, Wollstadt P, Lizier JT, Priesemann V. Quantifying Information Modification in Developing Neural Networks via Partial Information Decomposition. Entropy 2017;19:494.

[4] Timme N, Alford W, Flecker B, Beggs JM. Synergy, redundancy, and multivariate information measures: an experimentalist's perspective. J. Comput. Neurosci. 2014; 36:119-140. doi:10.1007/s10827-013-0458-4

[5] Ince RAA, Giordano BL, Kayser C, Rousselet GA, Gross J, Schyns PG. A Statistical Framework for Neuroimaging Data Analysis Based on Mutual Information Estimated via a Gaussian Copula. Hum. Brain Mapp. 2017;38:1541-1573.

[6] Park H, Ince RAA, Schyns PG, Thut G, Gross J. Representational interactions during audiovisual speech entrainment: Redundancy in left posterior superior temporal gyrus and synergy in left motor cortex. PLOS Biology 2018;16(8), e2006558. doi:10.1371/journal.pbio.2006558. PMID 30080855

[7] Wibral M, Lizier, JT, Priesemann, V. Bits from brains for biologically inspired computing. Frontiers in Robotics and AI 2015; 2. https://doi.org/10.3389/frobt.2015.00005

[8] Lizier JT, Bertschinger N, Jost J, Wibral M. Information Decomposition of Target Effects from Multi-Source Interactions: Perspectives on Previous, Current and Future Work. Entropy 2018;20(4):307. https://doi.org/10.3390/e20040307

[9] Kay JW, Ince RAA, Dering B Phillips WA. Partial and Entropic Information Decompositions of a Neuronal Modulatory Interaction. Entropy 2017;19(11):560. doi:10.3390/e19110560.

[10] Kay, JW, Phillips, WA Contextual Modulation in Mammalian Cortex is Asymmetric. Symmetry, 12, 815.

[11] McGill WJ. Multivariate Information Transmission. Psychometrika 1954; 19(2):97-116.

[12] Schneidman E, Bialek W, Berry MJ. Synergy, Redundancy, and Population Codes. J. Neurosci. 2003;23:11539-11553.

[13] Gat I, Tishby N. Synergy and redundancy among brain cells of behaving monkeys. In: Proceedings of the 1998 conference on Advances in neural information processing systems 2. Cambridge, MA, USA: MIT Press 1999. pp. 111-117.

[14] Kay J, Floreano D, Phillips WA. Contextually guided unsupervised learning using local multivariate binary processors. Neural Networks 1998;11;117-140.

[15] Timme NM, Lapish C. A Tutorial for Information Thoery in Neuroscience. eNeuro, https://doi.org/10.1523/ENEURO.0052-18.2018

[16] Ince RAA, Petersen RS, Swan DC, Panzeri S. Python for information theoretic analysis of neural data. Front. Neuroinform. 2009; 3(Art 4), (doi:10.3389/neuro.11.004.2009).

[17] Efron B, Tibshirani RJ. An Introduction to the Bootstrap. Chapman and Hall. New York. 1993.

[18] Stark E, Abeles,M. Applying resampling methods to neurophysiological data. Journal of Neuroscience Methods, 2009;145:133-144.

[19] Barrett DW, Gonzales-Lima F. Prefrontal-limbic functional connectivity during acquisition and extinction of conditioned fear. Neurosceince, 2018; 376:162-171.

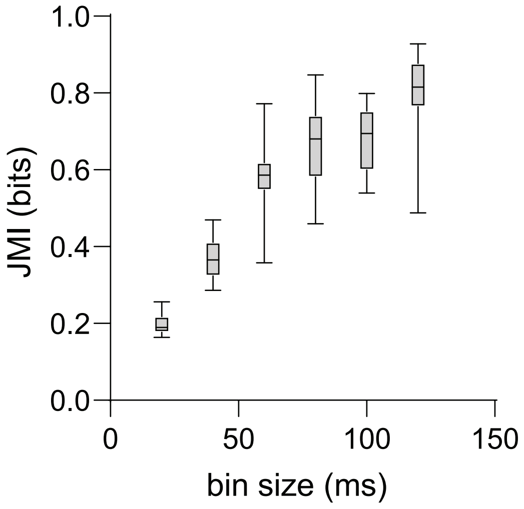

Figure S1. Dependence of joint mutual information on bin size of the PID analysis.
Data is shown for 16 neurons in control condition.

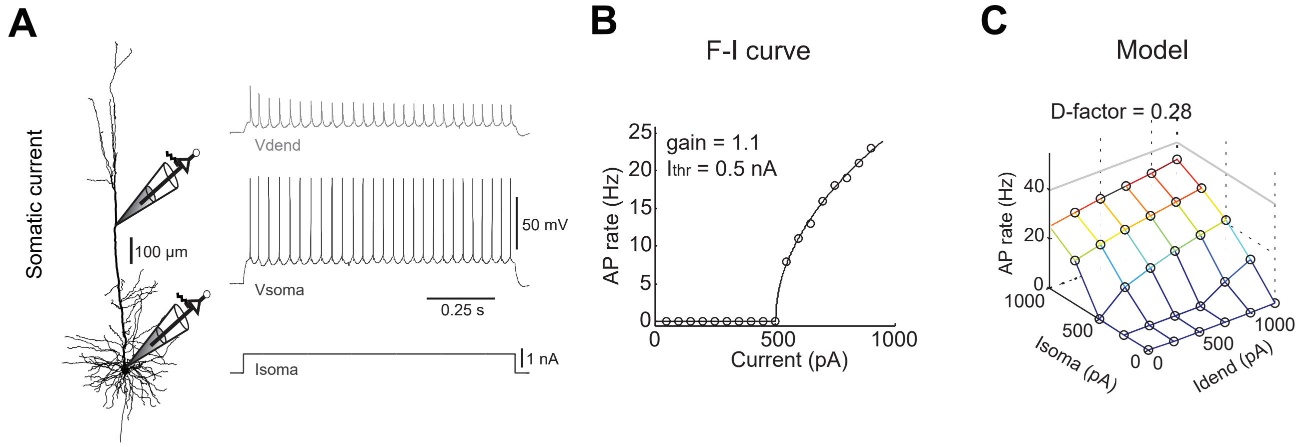

Figure S2. Extension of the spike rate model for dendritic current injections.
A) Left, locations of dual dendritic and somatic patch-clamp recordings are indicated on a biocytin-filled layer 5 pyramidal neuron. The distance between dendritic and somatic patch electrode was 500 µm. Right, dendritic and somatic membrane potential responses to somatic current injection. **B**) AP rate versus current (F-I) relationship for somatic current injections. Parameters of the fit of a square root function to the data of the same neuron are indicated. **C**) Extrapolation of the F-I relationship for combinations of dendritic and somatic current injections according to a passive dendrite spike rate model using eq. (*1*). Estimates of the *gain* and threshold current *Ithr* were derived from fits of AP rate data in response to increasing amplitudes of somatic current steps (B; 100 pA step size). The dendritic gain factor *D* was set to the transfer resistance normalized to the somatic input resistance (*D*=Rd,s/Rin) to capture the relative effect of dendritic current on the somatic membrane potential and, thus, spike output in the case of completely passive dendrites.

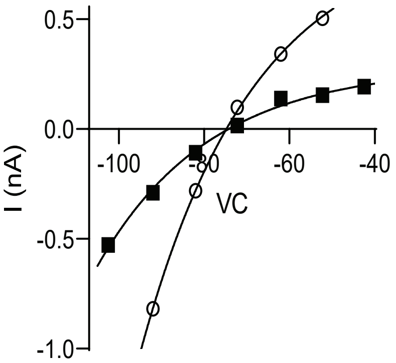

Figure S3. GIRK-mediated currents measured in CA3 pyramidal neurons from Fig.4B in Gähwiler & Brown (1985).
Open circle indicate baclofen induced currents, filled squares indicate GABA-induced currents in the presence of pitrazepin (Data was reproduced with permission by the authors from Gähwiler, B.H. & Brown, D.A. (1985) GABAB-receptor-activated K+ current in voltage-clamped CA3 pyramidal cells in hippocampal cultures. Proc Natl Acad Sci USA, 82, 1558-1562). Solid lines are fits of
I(v)= Scale·(v-E_K)·(g_min+ (g_max- g_min) / (1+e^((V_50-v)/Slope)))
to the data. For the fit, the following parameters were constrained: gmax=1; gmin=0; V50=EK=-74.5 mV.

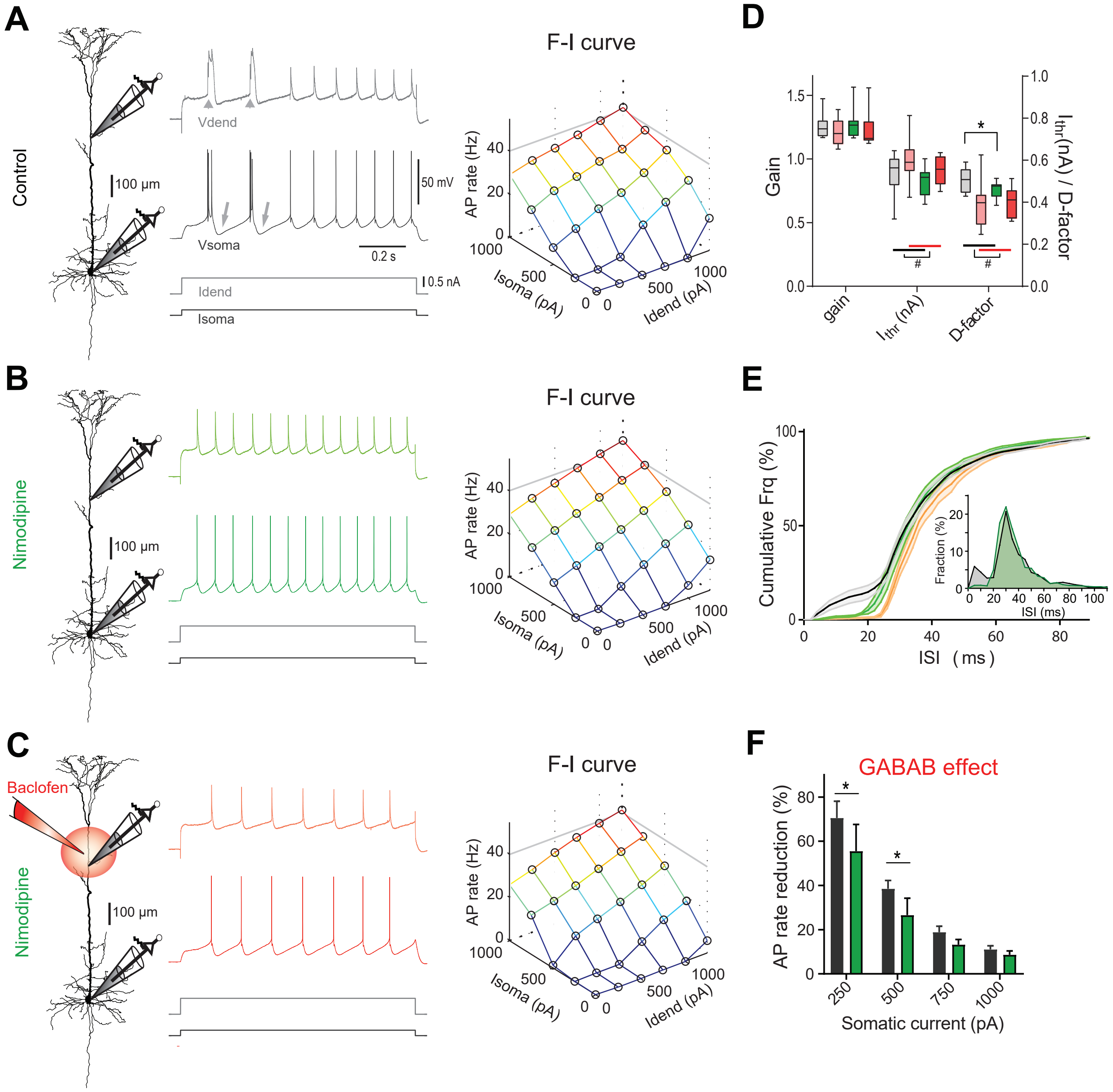

Figure S4. GABABR-mediated inhibition of L-type Ca2+ channels prevents burst firing. **A**) Left, locations of dual dendritic and somatic patch-clamp recordings on a biocytin-filled layer 5 pyramidal neuron. The distance between dendritic and somatic patch was 510 µm. Center, membrane potential responses of the same neuron to combined dendritic and somatic current injection. Dendritic Ca2+ spikes (arrowheads) and subsequent sAHPs (arrows) are indicated. Right, experimentally observed AP rate versus current (F-I) relationship for the same neuron. **B**) Membrane potential responses of the same neuron to combined dendritic and somatic current injection after pharmacological block of L-type Ca2+ channels by bath application of nimodipine (10 µM). Note the absence of dendritic Ca2+ spikes. **C**) Membrane potential responses and F-I relationship during a puff of baclofen in the presence of nimodipine. **D**) Parameters of the F-I relationship from fits to eq. (1) are shown for the control condition (gray), baclofen puff (light red), in the presence of nimodipine (green) and nimodipine in combination with a baclofen puff (red; n=7). The dendritic gain factor *D* was significantly decreased by the application of nimodipine (*, $P<0.01$, Wilcoxon signed rank test). Main effects of baclofen are indicated (#, $P<0.05$, ANOVA). **E**) Grand mean of the cumulative ISI distribution (n=7 neurons). In control condition (grey), there was a high proportion of fast ISIs (<20 ms), which were lost after wash-in of nimodipine (green). An additional puff of baclofen shifted the whole ISI distribution to the right (orange). Shaded area indicates the SEM. Inset shows the frequency distribution of all ISIs in control condition and after wash-in of nimodipine. Bin width was 5 ms. **F**) AP rate reduction during dendritic puff of baclofen in percent of baseline AP rate. Episodes of the same somatic current amplitudes were grouped across all dendritic current level. An ANOVA of the spike rate reduction after the baclofen puff showed a significant interaction between nimodipine and stimulation intensity ($P=0.017$, $F(3,18)=4.45$) but did not show a main effect of nimodipine ($P=0.12$, $F(1,6)=3.38$). Asterisks indicate significantly smaller reductions of the AP rate in the presence of nimodipine (green; $P<0.01$, Bonferroni's multiple comparisons test), indicating a partial occlusion of the baclofen effect.

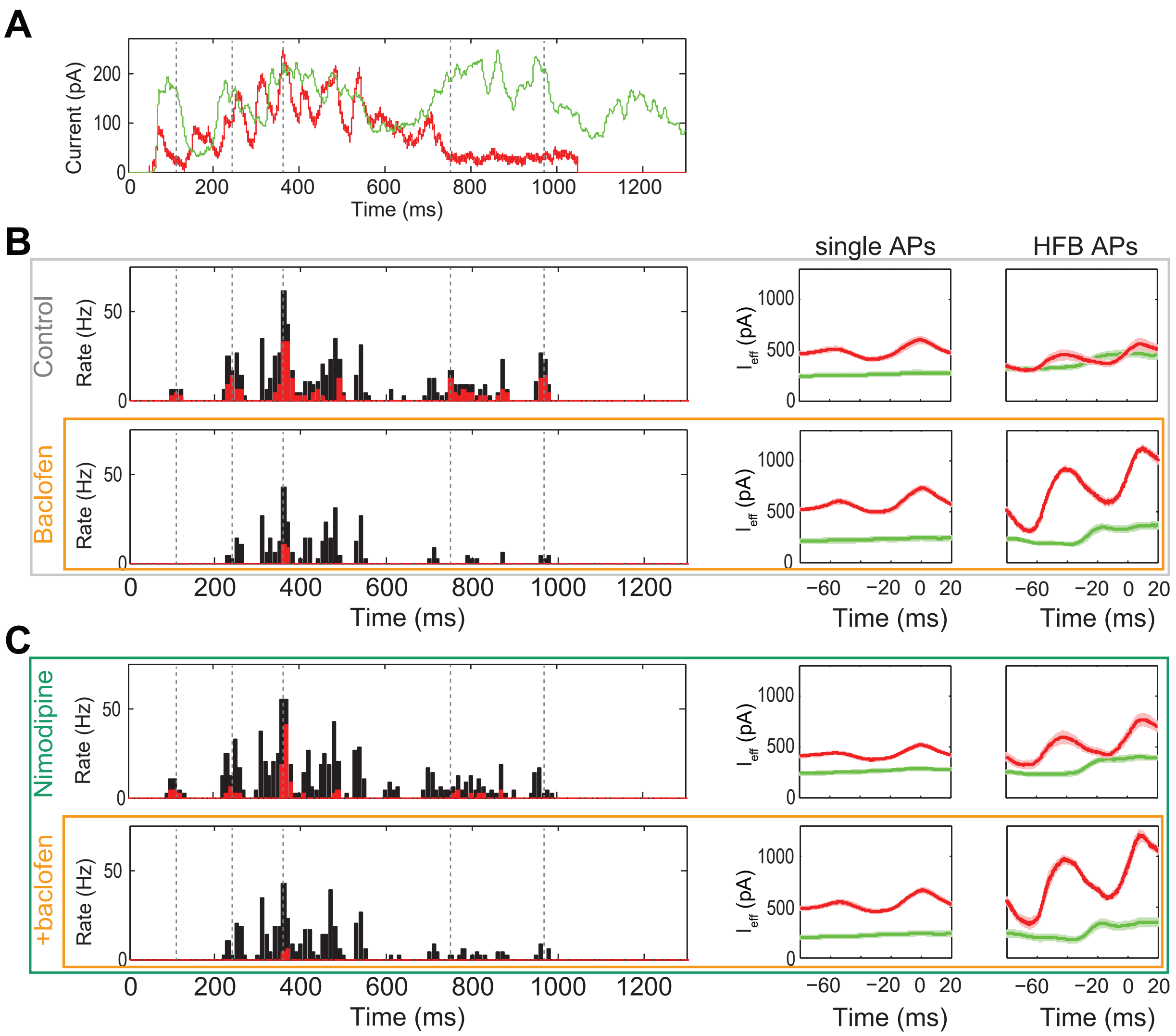

Figure S5. Differential regulation of single APs and HFBs during in vivo-like input waveforms by dendritic GABABRs.

A) Injected current waveforms based on responses to sensory stimulation of the contralateral hind limb recorded in vivo. Dendritic current is shown in green, somatic in red. **B**) Peri-stimulus time histograms (PSTH, left) of all APs (black) and APs that are part of a HFB (red) in a neuron during control condition (top) and while puffing baclofen onto the apical dendrite (bottom). Vertical dashed lines in the PSTH and the current waveforms (A) indicate time points of consistently reoccurring HFBs across episodes. On the right, the grand mean (n=6 neurons) of the dendritic (green) and the somatic (red) spike-triggered average (STA) of the effective current (Ieff) is shown. The number of APs detected per condition within individual neurons ranged between 71 to 249 for single APa (sAP) and 9 to 62 for HFBs in control; 66 to 216 for sAPs and 2 to 8 for HFBs during the baclofen puff. To obtain a more accurate estimate of the current that effectively drove the axonal AP output, the dendritic current was scaled by the dendritic gain factor *D*. *D* was obtained from the fit of eq. (*1*) to the F-I relationship during linear current step injections in the same neurons under the same pharmacological conditions. Note the different temporal modulation of somatic Ieff and the increased level of dendritic Ieff necessary for the induction of HFB compared to single APs during the control condition. Dendritic activation of GABABRs by puffed baclofen strongly increased the contribution of somatic Ieff, suggesting that AP output was mainly driven by the somatic current injection. **C**) The effect of the L-type Ca2+ channel blocker nimodipine on AP output. Left, PSTH from the same neuron before and during dendritic baclofen application in the presence of the L-type Ca2+ channel blocker nimodipine. Right, grand means of Ieff from the same group of neurons before and during baclofen application. The number of APs detected per condition within individual neurons ranged between 153 to 327 for sAPs and 9 to 38 for HFBs in nimodipine; 99 to 273 for sAPs and 1 to 6 for HFBs in nimodipine plus baclofen puff. Note the reduced contribution of dendritic Ieff and the increased contribution of somatic Ieff to HFBs in the presence of nimodipine.

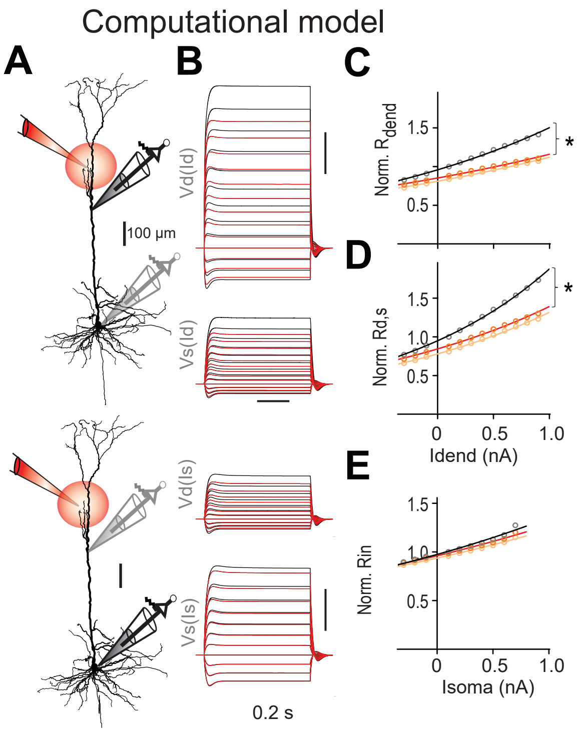

Figure S6. GABAB-mediated GIRK channel activation reduces the transfer resistance. **A**) A detailed multicompartmental NEURON model containing a realistic HCN channel density but no voltage-gated Ca2+ in the dendrites recapitulates the GABABR -mediated effect on Rin, Rdend and Rd,s. The model was based on the morphology (A) and recording data from the neuron shown in Fig.4A. **B**) GIRK channel-induced hyperpolarization was compensated by a constant current injection injection of +70.5 pA into the dendrite. GIRK-mediated shunt nonetheless significantly increased doubling interval of Rdend from 1.5 in control (black) to 2.3 nA ($P<0.0001$, $F(1,20)=130.5$; orange). Traces in lighter colors indicate results from the same model without the compensatory current injection. **C**) GIRK channel-mediated shunt increased the doubling interval from 1.0 to 1.4 nA of Rd,s ($P<0.0001$, $F(1,20)=199.3$). **D**) The voltage-sensitivity of the normalized somatic Rin was not changed significantly by the GIRK channel-mediated shunt ($P=0.11$, $F(1,16)=2.85$).

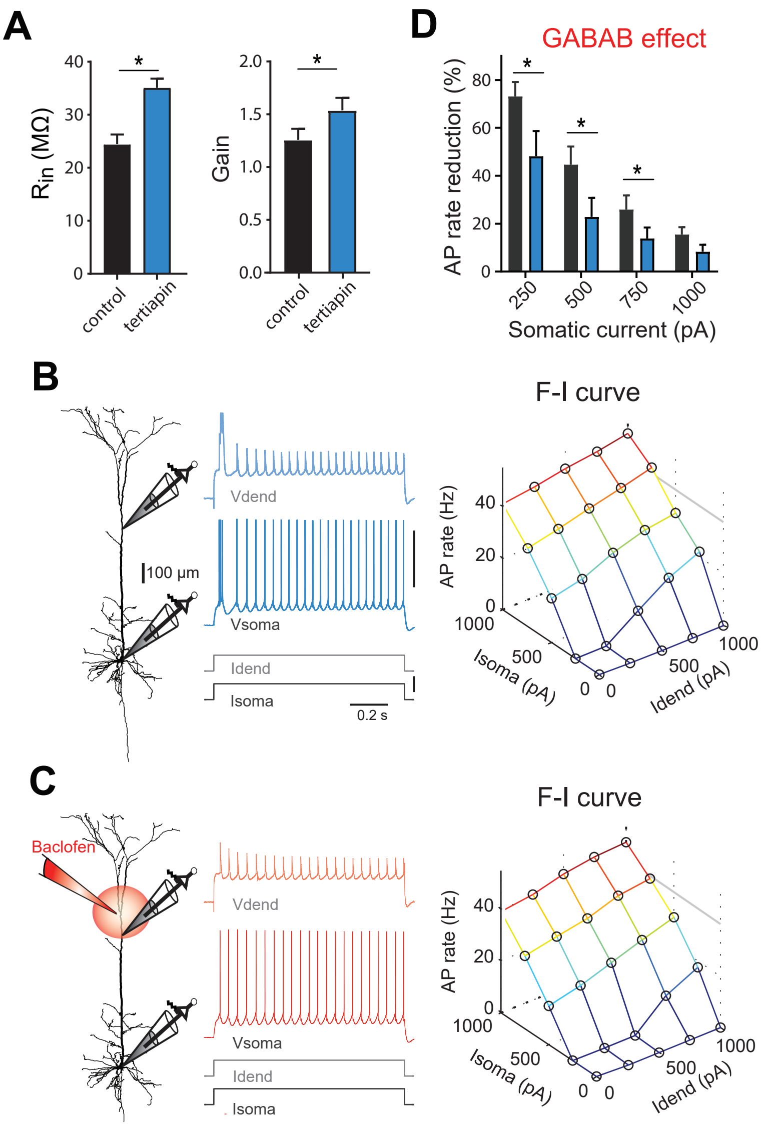

Figure S7. GIRK channel-activation in dendrites decreases action potential output. A) The somatic input resistance was significantly increased in the presence of tertiapin (P=0.016, n=7, Wilcoxon signed rank test) and resulted in an increased gain in the F-I relationship (P=0.016, n=7). **B**) Left, schematic of recording arrangement (dendritic to somatic patch distance=610 µm). Center, membrane potential responses to combined current injection in the presence of tertiapin (0.5 µM). Right, F-I relationship for the same neuron. **C**) Membrane potential responses and F-I relationship during a puff of baclofen in the presence of tertiapin. **D**) AP rate reduction during baclofen application in percent of baseline AP rate. Episodes of the same somatic current amplitudes were grouped across all dendritic current level. There was a main effect of tertiapin (blue; P=0.013, ANOVA, F(1,6)=12.14). Asterisks indicate significantly smaller reductions of the AP rate in the presence tertiapin (P<0.05, Bonferroni's multiple comparisons test), demonstrating a block of the baclofen effect.
